# Comparative Risks of High-Grade Adverse Events Among FDA-Approved Systemic Therapies in Advanced Melanoma: Systematic Review and Network Meta-Analysis

**DOI:** 10.3389/fonc.2020.571135

**Published:** 2020-10-15

**Authors:** Ya-fang Huang, Wen-jie Xie, Hai-yu Fan, Juan Du

**Affiliations:** ^1^School of General Practice and Continuing Education, Capital Medical University, Beijing, China; ^2^Department Clinical Research, University of Bern, Bern, Switzerland; ^3^Center of Stroke, Beijing Institute for Brain Disorders, Capital Medical University, Beijing, China

**Keywords:** immune checkpoint inhibitor (ICI), targeted inhibitor, network meta-analysis, advanced melanoma, high-grade adverse event

## Abstract

**Background:** Head-to-head evidence is lacking in comparative risks of high-grade adverse events (AEs) among different systemic treatment options for advanced melanoma.

**Methods:** An up-to-date systematic review and network meta-analysis (NMA) was performed. Randomized controlled trials (RCTs) of patients with advanced melanoma were eligible if at least one intervention was the Food and Drug Administration–approved targeted or immune checkpoint inhibitors. Risks of high-grade AEs were estimated by random-effects Bayesian NMAs, based on relative risks. Surface under the cumulative ranking probabilities was used to assess relative ranking of treatments. The summary incidences were calculated.

**Results:** Twenty-five RCTs (12,925 patients) comparing 10 different systemic treatment options were included. BRAF/MEK had the highest risk of overall high-grade AEs (pooled incidence: 32.11%). BRAF had the highest risk of high-grade arthralgia (0.39%), whereas MEK had the highest risk of high-grade hypertension (2.28%) and nausea (0.37%). Cytotoxic T-lymphocyte antigen 4 (CTLA-4)/chemo had the highest risk of high-grade diarrhea (1.31%), alanine aminotransferase (0.60%), and aspartate aminotransferase elevation (0.59%). Programmed cell death 1 (PD-1)/CTLA-4 had the highest risks of high-grade pyrexia (1.14%) and rash (0.94%). Using PD-1 inhibitor alone had the lowest risks of overall high-grade AEs.

**Conclusions:** Different systemic treatment options have varying high-grade AEs in advanced melanoma treatment. Current evidences highlight the important risks of BRAF/MEK, CTLA-4/chemo, and PD-1/CTLA-4.

## Introduction

Systemic therapy is the main treatment modality for patients with advanced melanoma (1). The landscape of systemic treatment options is changing rapidly in recent years from traditional interferon α to novel mitogen-activated protein kinase pathway inhibitors (i.e., BRAF inhibitors and MEK inhibitors) and immune checkpoint inhibitors (ICIs) [i.e., programmed cell death 1 inhibitors (PD-1) and cytotoxic T-lymphocyte antigen 4 inhibitors (CTLA-4)] (2). Results from randomized controlled trials (RCTs) have shown that these new agents have drastically improved progression-free survival (PFS) and overall survival (OS) in patients with advanced melanoma (3, 4). However, high-grade adverse events (AEs) related to these targeted inhibitors and ICIs remain a concern in clinical practice (1).

Medical decision-making for patients with advanced melanoma is a major challenge for clinicians. It is important to balance between the clinical benefits and potential high-grade risks of each systemic treatment option during decision making (2, 5). Systematic review and network meta-analysis (NMA) have been conducted to provide high-quality evidences to support the medical decision-making. For example, previous studies found BRAF plus MEK combination was the most favorable therapy to improve PFS, whereas PD-1 was associated with improved OS benefit (5–9). However, these studies were mainly focused on the comparative efficacy. The risks of severe, life-threatening AEs or deaths related to the BRAF- or MEK-targeted inhibitors or ICIs treatments were not adequately summarized for patients with advanced melanoma (6, 9).

The decision about systemic therapies to patients with advanced melanoma should be informed not only by the reduction of recurrence risk or OS improvement, but also by careful management of high-grade risks (10). In the absence of a direct comparison among different systemic treatment options to guide the clinical decision-making, it has been unclear which treatment strategy has the highest high-grade AEs to patients with advanced melanoma. A comprehensive understanding of the high-grade AEs of these novel targeted and immunotherapy agents is needed for informed these clinical decisions. We conducted a NMA to compare high-grade AEs of the Food and Drug Administration (FDA)–approved ICIs and targeted inhibitors for patients with advanced melanoma.

## Materials and Methods

### Study Design

This NMA was reported based on the Preferred Reporting Items for Systematic Reviews and Meta-Analyses guidelines (11, 12). *A priori* established review protocol was followed when the study was conducted. The review protocol was registered in the PROSPERO international prospective register of systematic reviews (CRD42020160453).

### Search Strategy and Selection Criteria

The final searches of PubMed, EMBASE, and Cochrane Library were conducted up to December 20, 2019, using the combinations of the following terms: (melanoma OR melanocyte) AND (ipilimumab OR yervoy OR nivolumab OR opdivo OR pembrolizumab OR keytruda OR binimetinib OR mektovi OR cobimetinib OR cotellic OR dabrafenib OR tafinlar OR encorafenib OR braftovi OR trametinib OR mekinist OR vemurafenib OR zelboraf OR “cytotoxic T-lymphocyte antigen 4” OR “programmed cell death 1 receptor” OR “BRAF” OR “MEK”) AND (random OR control OR phase II OR phase III OR placebo) without restriction on year of publication or language. The detailed search strategies are listed in Supplementary Table 1.

Trials were eligible if the following inclusion criteria were met: (1) patients with advanced melanoma regardless mutation status; (2) at least one of the interventions compared in the trial was either the FDA-approved ipilimumab, nivolumab, pembrolizumab, binimetinib, cobimetinib, dabrafenib, encorafenib, trametinib, vemurafenib, their combinations, or chemotherapy with their combinations; (3) high-grade AEs were extractable either from published articles or unpublished reports from clinicaltrial.gov; (4) phase II or III RCTs. We excluded (1) commentaries, letters, editorials, protocols or reviews; (2) trials only in conference abstracts/posters form; (3) phase I, dose escalation or single-arm trials; (4) *in vitro* or animal studies; and (5) studies of cost-effectiveness analyses or quality of life. The titles, abstracts and full texts were evaluated sequentially.

### Data Extraction

Data from eligible trials were extracted by two investigators (HY and FH). The extracted information included trial name, line of treatment, study phase, blinding status, median age (range), sex, mutation status, resection status, treatment class [BRAF, MEK, BRAF, and MEK combination (BRAF/MEK), CTLA-4, PD-1, chemotherapy, PD-1 and CTLA-4 combination (PD-1/CTLA-4), CTLA-4, and chemotherapy combination (CTLA-4/chemo)], dosage of drugs, number of patients in each randomization arm, median length of follow-up in each treatment arm, number of patients in the safety dataset, and number of patients with the following: [1] overall high-grade AEs (grades 3–5 AEs); [2] general symptomatic high-grade AEs (fatigue, pyrexia); [3] general laboratory results–related high-grade AEs [alanine aminotransferase (ALT) elevation, aspartate aminotransferase (AST) elevation, hypertension]; [4] musculoskeletal/pain–related high-grade AEs (arthralgia, myalgia); [5] gastrointestinal high-grade AEs (diarrhea, nausea); and [6] cutaneous high-grade AEs (rash). Both published data from articles and unpublished data from clinicaltrial.gov were extracted. When discrepancies occurred between the published and unpublished data, we selected the data with higher number of events.

### Quality Assessment

The risk of bias was assessed by two authors (HY and FH) independently. The domains assessed included random sequence generation (selection bias), allocation concealment (selection bias), blinding of participants and personnel (performance bias), blinding of outcome assessment (detection bias), incomplete outcome data (attrition bias), selective reporting (reporting bias), and other bias (13).

### Outcome Measures

The primary outcome was the incidence of overall high-grade AEs. The secondary outcome was the incidence of general symptomatic high-grade AEs (fatigue, pyrexia), general laboratory results–related high-grade AEs (ALT/AST elevation, hypertension), musculoskeletal/pain–related high-grade AEs (arthralgia, myalgia), gastrointestinal high-grade AEs (diarrhea, nausea), and cutaneous high-grade AEs (rash). Both the primary and secondary outcomes were defined as grades 3–5 AEs basing on the National Cancer Institute Common Terminology Criteria for Adverse Events (CTCAE) version 4.0.

### Data Synthesis and Statistical Analysis

NMA was conducted based on the Bayesian framework using a Markov Chain Monte Carlo (MCMC) simulation technique. Non-informative priors were used to estimate the posterior distribution (14). The MCMC model was updated with 100,000 simulated draws after a burn-in of 20,000 iterations. We used a thinning interval of 10 for each chain. Brooks–Gelman–Rubin statistic was used to assess the adequacy of burn-in and convergence (15). Relative risks (RRs) along with corresponding 95% credible intervals were reported. Random-effects model was used because they generally show better goodness of fit. The posterior mean of the residual deviance was calculated to assess goodness of model fit. The incidence of both primary and secondary outcomes was estimated (incidence = 100 × assumed placebo risk × RR, the assumed placebo risk was generated by using traditional meta-analyses with random-effects model).

Hierarchy of both the primary and secondary outcomes was respectively estimated for all the treatment classes using median ranks and surface under the cumulative ranking curve (SUCRA). SUCRA was the percentage of drug safety on AEs that would be ranked first without uncertainty. When the drug safety was certain to be the best, the SUCRA value would equal one, whereas it would equal zero when the safety was certain to be the worst (16). The presence of inconsistency was evaluated by node splitting analysis in the entire network on particular comparisons (17, 18). The *P* < 0.05 was regarded as significant inconsistency. All the data analyses were conducted using STATA version 14.0 and WinBUGs version 1.4.3.

## Results

### Selection of Trials

Initially, 2,955 unduplicated records were identified by literature search. After screening of titles and abstracts, 2,895 records were excluded. Sixty articles were assessed for eligibility. Finally, 49 articles involving 25 RCTs were included for qualitative and quantitative synthesis (3, 4, 19–65) (Supplementary Figure 1).

### Characteristics of Trials and Patients

The 25 RCTs covered 10 treatment classes and included 12,925 patients with advanced melanoma (Table 1 and Supplementary Table 2). Supplementary Table 3 lists the arrangement of treatments into treatment classes. Among the 25 RCTs, 19 trials (76.0%) were phase III studies, and 15 trials (60.0%) included patients with first-line treatment. The median age of patients was between 50 and 65 years. Supplementary Table 4 shows the details of risk-of-bias assessment based on each trial.

**Table 1:** Characteristics of included trials (49 articles including 25 randomized controlled trials).

**Trial name**	**Line of treatment**	**Study phase**	**Blinding**	**Median age (range)**	**Sex (Male)**	**Mutation status**	**Resection status**	**Treatment class**	**Treatment**	**Follow up (month)**	**No of patients in safety dataset**	**No of patients with grades 3–5 AEs**
BREAK-3	First-line	Phase 3	Open-label	52 (21–93)	149	BRAF V600E mutation	Unresectable	BRAF	Dabrafenib 150 mg twice daily (187)	NA	187	64^*^
								Chemotherapy	Dacarbazine 1,000 mg/m^2^ every 3 weeks (63)	NA	59	14^*^
BRF113220	First-line	Phase 2	Open-label	50 (18–85)	93	BRAF V600E or V600K mutations	Unresectable	BRAF/MEK	Trametinib 1 mg once daily plus dabrafenib 150 mg twice daily (54)^**^	Median 14.1	54	30
								BRAF/MEK	Trametinib 2 mg once daily plus dabrafenib 150 mg twice daily (54)	Median 14.1	55	42
								BRAF	Dabrafenib 150 mg twice daily (54)	Median 14.1	53	25
BRIM-3	First-line	Phase 3	Open-label	54 (17–86)	381	BRAF V600E mutation	Unresectable	BRAF	Vemurafenib 960 mg twice daily (337)	Median 13.4	336	165
								Chemotherapy	Dacarbazine 1,000 mg/m^2^ every 3 weeks (338)	Median 9.2	293	52
BRIM-8	First-line	Phase 3	Double-blind	51 (38–61)	283	BRAF V600E mutation	Resected	BRAF	Vemurafenib 960 mg twice daily (250)	Median 30.8 in cohort 1; Median 33.5 in cohort 2	247	142
								Placebo	Placebo (248)	Median 30.8 in cohort 1; Median 33.5 in cohort 2	247	37
CA184-004	Not clear	Phase 2	Double-blind	55 (23–87)	52	Not clear	Unresectable	CTLA-4 low dose	Ipilimumab at 3 mg/kg every 3 weeks (40)	Median 8.9	40	7
								CTLA-4 high dose	Ipilimumab at 10 mg/kg every 3 weeks (42)	Median 8.6	42	14
CA184-022	Not clear	Phase 2	Double-blind	59 (19–85)	144	Not clear	Unresectable	CTLA-4	Ipilimumab 0.3 mg/kg every 3 weeks (73)^**^	Median 8.3	72	26
								CTLA-4 low dose	Ipilimumab 3 mg/kg every 3 weeks (72)	Median 8.7	71	35
								CTLA-4 high dose	Ipilimumab 10 mg/kg every 3 weeks (72)	Median 10.7	71	38
CA184-024	Not clear	Phase 3	Double-blind	57 (31–87)	301	Not clear	Unresectable	CTLA-4 plus chemotherapy	Ipilimumab 10 mg/kg plus dacarbazine 850 mg/m^2^ (250)	Range: 36.6–54.0	247	170^*^
								Chemotherapy	Dacarbazine 850 mg/m^2^ every 3 weeks (252)	Range: 36.6–54.0	251	121^*^
CA184-169	First-line	Phase 3	Double-blind	62 (49–71)	450	BRAF V600E,V600K, other mutation, or wild type	Unresectable	CTLA-4 high dose	Ipilimumab 10 mg/kg every 3 weeks (365)	Median 14.5	364	245^*^
								CTLA-4 low dose	Ipilimumab 3 mg/kg every 3 weeks (362)	Median 11.2	362	194^*^
CheckMate 037	Second-line	Phase 3	Open-label	60 (23–85)	261	BRAF V600E, V600K, or wild type	Unresectable	PD-1	Nivolumab 3 mg/kg every 2 weeks (272)	Median 8.4	268	156^*^
								Chemotherapy	Dacarbazine 1,000 mg/m^2^ every 3 weeks or carboplatin AUC = 6 plus paclitaxel 175 mg/m^2^ every 3 weeks (133)	Median 8.4	102	46
CheckMate 066	First-line	Phase 3	Double-blind	65 (18–87)	246	Wild type	Unresectable	PD-1	Nivolumab 3 mg/kg every 2 weeks (210)	Median 8.9	206	70
								Chemotherapy	Dacarbazine 1,000 mg/m^2^ every 3 weeks (208)	Median 6.8	205	78
CheckMate 067	First-line	Phase 3	Double-blind	60 (18–90)	610	BRAF V600E, V600K, or wild type	Unresectable	PD-1	Nivolumab 3 mg/kg every 2 weeks (316)	Median 35.7	313	188
								CTLA-4 plus PD-1	Nivolumab 1 mg/kg every 3 weeks plus ipilimumab 3 mg/kg every 3 weeks (314)	Median 38.0	313	223^*^
								CTLA-4 low dose	Ipilimumab 3 mg/kg every 3 weeks (315)	Median 18.6	311	173
CheckMate 069	First-line	Phase 2	Double-blind	65 (27–87)	95	Not clear	Unresectable	CTLA-4 plus PD-1	Nivolumab 1 mg/kg plus ipilimumab 3 mg/kg every 3 weeks (95)	Minimum 11	94	58^*^
								CTLA-4 low dose	Ipilimumab 3 mg/kg every 3 weeks (47)	Minimum 11	46	18^*^
CheckMate 238	Not clear	Phase 3	Double-blind	55 (18–86)	527	BRAF V600E, V600K, or wild type	Resected	PD-1	Nivolumab 3 mg/kg every 2 weeks (453)	Median 19.5	452	115
								CTLA-4 high dose	Ipilimumab 10 mg/kg every 3 weeks (453)	Median 19.5	453	252
coBRIM	First-line	Phase 3	Double-blind	55 (23–88)	286	BRAF V600E mutation	Unresectable	BRAF/MEK	Vemurafenib 960 mg twice daily plus cobimetinib 60 mg once daily (247)	Median 7.3	247	186
								BRAF	Vemurafenib 960 mg twice daily (248)	Median 7.3	246	151
COLUMBUS	First-line	Phase 3	Open-label	56 (20–89)	334	BRAF V600E or V600K mutations	Unresectable	BRAF/MEK	Encorafenib 450 mg once daily plus binimetinib 45 mg twice daily (192)	Median 16.7	192	112
								BRAF	Encorafenib 300 mg once daily (194)^**^	Median 16.6	192	127
								BRAF	Vemurafenib 960 mg twice daily (191)	Median 14.4	186	118
COMBI-AD	First-line	Phase 3	Double-blind	50 (18–89)	388	BRAF V600E or V600K mutations	Resected	BRAF/MEK	Dabrafenib 150 mg twice daily plus trametinib 2 mg once daily (438)	Median 33.6	435	181
								Placebo	Placebo (432)	Median 33.6	432	61
COMBI-d	First-line	Phase 3	Double-blind	56 (22–89)	225	BRAF V600E or V600K mutations	Unresectable	BRAF/MEK	Dabrafenib 150 mg twice daily plus trametinib 2 mg once daily (211)	Median 9	209	104
								BRAF	Dabrafenib 150 mg twice daily (212)	Median 9	211	106
COMBI-v	First-line	Phase 3	Open-label	55 (18–91)	388	BRAF V600E mutation	Unresectable	BRAF/MEK	Dabrafenib 150 mg twice daily plus trametinib 2 mg once daily (352)	Median 11	350	173
								BRAF	Vemurafenib 960 mg twice daily (352)	Median 10	349	206
EORTC 18071	First-line	Phase 3	Double-blind	52 (18–84)	589	Not clear	Resected	CTLA-4 high dose	Ipilimumab 10 mg/kg every 3 weeks (475)	Median 63.6	471	260
								Placebo	Placebo (476)	Median 64.8	474	124
KEYNOTE-002	Second-line or more	Phase 2	Open-label	62 (15–89)	327	BRAF V600E, V600K, or wild type	Unresectable	PD-1	Pembrolizumab 2 mg/kg every 3 weeks (180)^**^	Median 10	178	94^*^
								PD-1	Pembrolizumab 10 mg/kg every 3 weeks (181)	Median 10	179	78^*^
								Chemotherapy	Paclitaxel plus carboplatin, paclitaxel, carboplatin, dacarbazine, or oral temozolomide (179)	Median 10	171	45
KEYNOTE-006	First-line or second-line	Phase 3	Open-label	62 (18–89)	497	BRAF V600E, V600K, or wild type	Unresectable	PD-1	Pembrolizumab 10 mg/kg every 2 weeks (279)^**^	Median 22.9	278	90^*^
								PD-1	Pembrolizumab 10 mg/kg every 3 weeks (277)	Median 22.9	277	84^*^
								CTLA-4 low dose	Ipilimumab 3 mg/kg every 3 weeks (278)	Median 22.9	256	81^*^
KEYNOTE-054	Second-line or more	Phase 3	Double-blind	54 (19–88)	628	BRAF V600E, V600K, other mutation, or wild type	Resected	PD-1	Pembrolizumab 200 mg every 3 weeks (514)	Median 15	509	161
								Placebo	Placebo (505)	Median 15	502	104^*^
MDX010-08	Not clear	Phase 2	Open-label	61 (25–82)	47	Not clear	Unresectable	CTLA-4 plus chemotherapy	Ipilimumab 3 mg/kg every 4 weeks plus dacarbazine 250 mg/m^2^ every 3 weeks (36)	Median 20.9	35	9
								CTLA-4 low dose	Ipilimumab 3 mg/kg every 4 weeks (40)	Median 16.4	39	6
METRIC	Not clear	Phase 3	Open-label	54 (21–85)	173	BRAF V600E or V600K mutations	Unresectable	MEK	Trametinib 2 mg once daily (214)	Median 14.7	211	115
								Chemotherapy	Dacarbazine 1,000 mg/m^2^ every 3 weeks or carboplatin AUC = 6 or paclitaxel 175 mg/m^2^ every 3 weeks (108)	Median 8.7	99	40
NEMO	First-line	Phase 3	Open-label	64 (18–90)	251	NRAS mutation	Unresectable	MEK	Binimetinib 45 mg twice daily (269)	Median 1.7	269	91
								Chemotherapy	Dacarbazine 1,000 mg/m^2^ every 3 weeks (133)	Median 1.7	114	25

*AEs, adverse events; CTLA-4, cytotoxic T-lymphocyte-associated antigen-4 inhibitors; NA, not available; PD-1, programmed cell death protein 1 inhibitors*.

* *Data were extracted from clinicaltrial.gov*.

** *The treatment was not included in the network meta-analysis*.

### Overall High-Grade AEs

**Figure 1:**
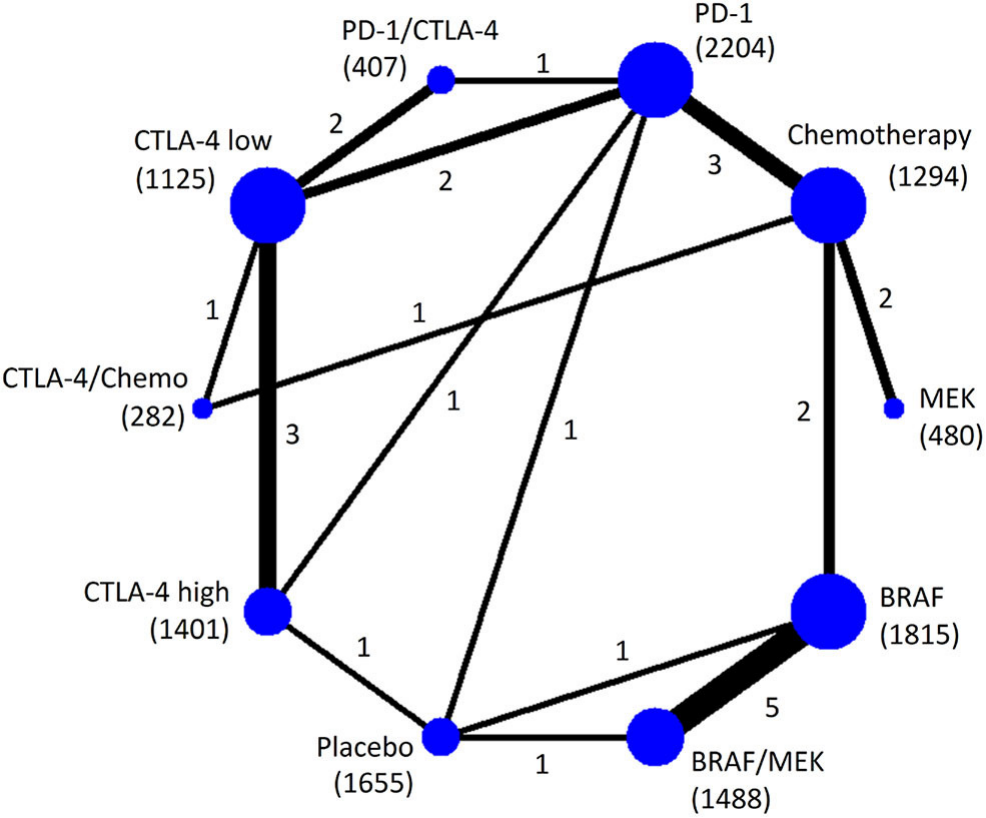
Network plot of eligible comparisons for the Bayesian network meta-analysis of overall high-grade AEs. The size of the nodes is proportional to the number of trials that involved the connected treatment (nodes). The width of the lines is proportional to the number of comparisons (beside the line) comparing the connected treatment (nodes). The number of patients randomized to receive the treatment is in parentheses. A total of 27 comparisons were analyzed for overall high-grade AEs.

Twenty-five RCTs (*n* = 12,151) were involved in the NMA of overall high-grade AEs (Figure 1). Pooled incidence was highest for BRAF/MEK (incidence = 32.11%, 95% CrI = 28.25–34.68%, SUCRA = 5.5%), followed by using BRAF alone (incidence = 31.50%, 95% CrI = 27.51–34.12%, SUCRA = 9.8%). Among the therapeutic treatments, the pooled incidence of overall high-grade AEs was lowest for using chemotherapy alone (incidence = 22.21%, 95% CrI = 16.02–27.95%, SUCRA = 86.0%), followed by using PD-1 inhibitors alone (incidence = 24.70%, 95% CrI = 19.17–29.49%, SUCRA = 71.9%) (Table 2).

**Table 2:** Median ranks and the pooled incidences of treatments in terms of high-grade AEs.

**Types**	**Treatment**	**Rank (95% CrIs)**	**SUCRA**	**Incidence (95% CrIs)**
**OVERALL HIGH-GRADE AEs**				
	Placebo	1 (1–3)	99.0	19.00%
	Chemo	2 (1–4)	86.0	22.21% (16.02–27.95%)
	PD-1	3 (2–6)	71.9	24.70% (19.17–29.49%)
	CTLA-4 low dose	4 (2–6)	65.5	25.37% (18.88–30.59%)
	MEK	5 (2–9)	49.4	27.28% (18.68–33.17%)
	CTLA-4/chemo	7 (3–10)	44.6	29.53% (21.45–34.45%)
	PD-1/CTLA-4	7 (4–10)	38.5	30.31% (23.37–34.56%)
	CTLA-4 high dose	7 (5–10)	29.7	30.46% (25.67–33.71%)
	BRAF	9 (5–10)	9.8	31.50% (27.51–34.12%)
	BRAF/MEK	9 (6–10)	5.5	32.11% (28.25–34.68%)
**GENERAL, SYMPTOMATIC HIGH-GRADE AEs**				
Fatigue	Placebo	1 (1–4)	91.4	0.50%
	CTLA-4 low dose	2 (1–4)	85.0	0.56% (0.27–0.82%)
	PD-1	2 (1–4)	83.6	0.58% (0.32–0.80%)
	CTLA-4 high dose	4 (2–6)	71.1	0.66% (0.43–0.84%)
	Chemo	6 (5–8)	43.9	0.86% (0.65–0.96%)
	PD-1/CTLA-4	6 (4–10)	37.8	0.87% (0.60–0.97%)
	MEK	7 (4–10)	37.3	0.88% (0.60–0.98%)
	BRAF	8 (5–10)	25.6	0.91% (0.77–0.98%)
	BRAF/MEK	9 (6–10)	13.0	0.93% (0.82–0.98%)
	CTLA-4/chemo	9 (6–10)	11.2	0.94% (0.78–0.99%)
Pyrexia	MEK	1 (1–5)	94.4	0.30% (0.03–0.95%)
	Placebo	3 (1–5)	82.8	0.60%
	Chemo	3 (1–5)	78.5	0.59% (0.18–0.98%)
	PD-1	4 (2–6)	71.2	0.72% (0.31–1.04%)
	BRAF	5 (2–7)	58.2	0.86% (0.42–1.10%)
	CTLA-4 low dose	7 (5–9)	33.4	1.08% (0.74–1.18%)
	CTLA-4/chemo	7 (4–10)	32.3	1.07% (0.58–1.19%)
	BRAF/MEK	8 (6–10)	19.8	1.13% (0.95%−1.18%)
	CTLA-4 high dose	9 (6–10)	15.9	1.14% (0.92–1.19%)
	PD-1/CTLA-4	9 (6–10)	13.5	1.14% (0.86–1.19%)
**GENERAL, LABORATORY HIGH-GRADE AEs**				
ALT elevation	Placebo	1 (1–3)	98.3	0.30%
	Chemo	3 (1–6)	76.3	0.51% (0.16–0.59%)
	PD-1	5 (2–7)	62.6	0.56% (0.38–0.60%)
	BRAF	4 (2–8)	62.5	0.55% (0.45–0.59%)
	MEK	4 (1–9)	60.8	0.55% (0.20–0.60%)
	CTLA-4 low dose	5 (2–8)	56.6	0.57% (0.37–0.60%)
	BRAF/MEK	6 (3–9)	42.7	0.57% (0.50–0.60%)
	CTLA-4 high dose	8 (6–10)	19.0	0.59% (0.55–0.60%)
	PD-1/CTLA-4	9 (6–10)	15.1	0.59% (0.55–0.60%)
	CTLA-4/chemo	10 (6–10)	6.2	0.60% (0.55–0.60%)
AST elevation	Placebo	1 (1–4)	94.6	0.30%
	Chemo	3 (1–6)	76.5	0.46% (0.06–0.59%)
	CTLA-4 low dose	4 (1–7)	71.1	0.51% (0.16–0.60%)
	PD-1	5 (2–7)	62.9	0.53% (0.26%−0.60%)
	BRAF	5 (2–8)	59.0	0.54% (0.37–0.59%)
	MEK	6 (2–10)	49.2	0.57% (0.15%−0.60%)
	BRAF/MEK	7 (3–10)	35.9	0.57% (0.47–0.60%)
	PD-1/CTLA-4	8 (5–10)	20.5	0.59% (0.47–0.60%)
	CTLA-4 high dose	9 (6–10)	15.6	0.59% (0.53–0.60%)
	CTLA-4/chemo	9 (4–10)	14.8	0.59% (0.39–0.60%)
Hypertension	Placebo	2 (1–7)	77.3	1.30%
	PD-1	4 (1–8)	65.0	1.66% (0.24–2.54%)
	Chemo	4 (1–8)	62.7	1.74% (0.22–2.54%)
	CTLA-4 low dose	4 (1–9)	61.4	1.68% (0–2.60%)
	BRAF	5 (2–9)	51.2	1.96% (1.14–2.46%)
	CTLA-4 high dose	8 (1–10)	40.7	2.39% (0.01–2.60%)
	PD-1/CTLA-4	8 (1–10)	38.2	2.37% (0.20–2.60%)
	BRAF/MEK	6 (2–10)	37.1	2.06% (1.28–2.46%)
	CTLA-4/chemo	8 (1–10)	36.1	2.40% (0.20–2.60%)
	MEK	7 (3–10)	30.3	2.28% (0.54–2.59%)
**MUSCULOSKELETAL/PAIN RELATED HIGH-GRADE AEs**				
Arthralgia	Placebo	1 (1–4)	94.4	0.20%
	PD-1	4 (2–8)	64.8	0.34% (0.18–0.39%)
	CTLA-4 high dose	4 (1–9)	59.2	0.34% (0.15–0.39%)
	Chemo	4 (2–8)	58.8	0.35% (0.18–0.39%)
	PD-1/CTLA-4	6 (1–10)	50.1	0.36% (0.13–0.40%)
	MEK	7 (1–10)	47.6	0.38% (0.07–0.40%)
	BRAF/MEK	6 (2–9)	40.7	0.37% (0.26–0.40%)
	CTLA-4 low dose	6 (2–10)	40.5	0.37% (0.18–0.40%)
	CTLA-4/chemo	9 (1–10)	32.2	0.39% (0.13–0.40%)
	BRAF	9 (5–10)	11.6	0.39% (0.34–0.40%)
Myalgia	PD-1/CTLA-4	1 (1–6)	82.8	NE
	CTLA-4 low dose	3 (1–8)	60.0	NE
	CTLA-4 high dose	3 (1–8)	59.3	NE
	Placebo	5 (1–7)	57.8	NE
	PD-1	4 (1–8)	54.4	NE
	Chemo	5 (2–8)	34.9	NE
	BRAF/MEK	7 (2–8)	34.1	NE
	BRAF	8 (3–8)	16.8	NE
**GASTROINTESTINAL HIGH-GRADE AEs**				
Diarrhea	MEK	2 (1–8)	84.6	0.56% (0.10–1.21%)
	PD-1	3 (1–5)	81.9	0.66% (0.34–1.02%)
	Chemo	3 (1–6)	78.6	0.66% (0.24–1.10%)
	Placebo	3 (1–6)	78.2	0.70%
	BRAF	5 (1–8)	55.3	0.93% (0.49–1.25%)
	CTLA-4 low dose	6 (4–8)	43.0	1.02% (0.66–1.25%)
	PD-1/CTLA-4	7 (4–9)	32.6	1.08% (0.66–1.30%)
	BRAF/MEK	8 (5–10)	23.9	1.18% (0.79–1.35%)
	CTLA-4 high dose	9 (7–10)	13.7	1.21% (1.02–1.33%)
	CTLA-4/chemo	10 (5–10)	8.3	1.31% (0.86–1.39%)
Nausea	Placebo	1 (1–5)	92.7	0.20%
	PD-1	3 (1–6)	78.3	0.28% (0.12–0.38%)
	CTLA-4 high dose	3 (1–9)	66.6	0.30% (0.14–0.38%)
	CTLA-4 low dose	4 (1–8)	63.5	0.30% (0.13–0.39%)
	BRAF	5 (2–9)	51.5	0.34% (0.19–0.39%)
	CTLA-4/chemo	6 (2–10)	46.4	0.34% (0.16–0.39%)
	Chemo	8 (4–10)	26.7	0.36% (0.24–0.39%)
	BRAF/MEK	8 (3–10)	25.9	0.36% (0.25–0.40%)
	PD-1/CTLA-4	8 (3–10)	24.4	0.37% (0.22–0.40%)
	MEK	9 (3–10)	23.9	0.37% (0.22–0.40%)
**CUTANEOUS HIGH-GRADE AEs**				
Rash	Chemo	1 (1–4)	93.2	0.26% (0.03–0.81%)
	Placebo	2 (1–6)	79.1	0.50%
	PD-1	4 (1–7)	70.6	0.65% (0.18–0.95%)
	BRAF/MEK	5 (2–9)	54.5	0.75% (0.29–0.96%)
	CTLA-4 low dose	6 (2–9)	48.9	0.81% (0.28–0.98%)
	CTLA-4/chemo	6 (1–10)	45.6	0.82% (0.14–1.00%)
	CTLA-4 high dose	7 (4–10)	31.7	0.87% (0.45–0.99%)
	MEK	8 (2–10)	29.9	0.90% (0.21–1.00%)
	BRAF	8 (4–10)	27.5	0.88% (0.57–0.98%)
	PD-1/CTLA-4	9 (4–10)	19.0	0.94% (0.49–1.00%)

*AEs, adverse events; ALT, alanine aminotransferase; AST, aspartate aminotransferase; CrIs, credible intervals; Chemo, Chemotherapy; CTLA-4, cytotoxic T-lymphocyte-associated antigen-4 inhibitors; NE, not estimable; PD-1, programmed cell death protein 1 inhibitors; SUCRA, surface under the cumulative ranking curve*.

**Figure 2:**
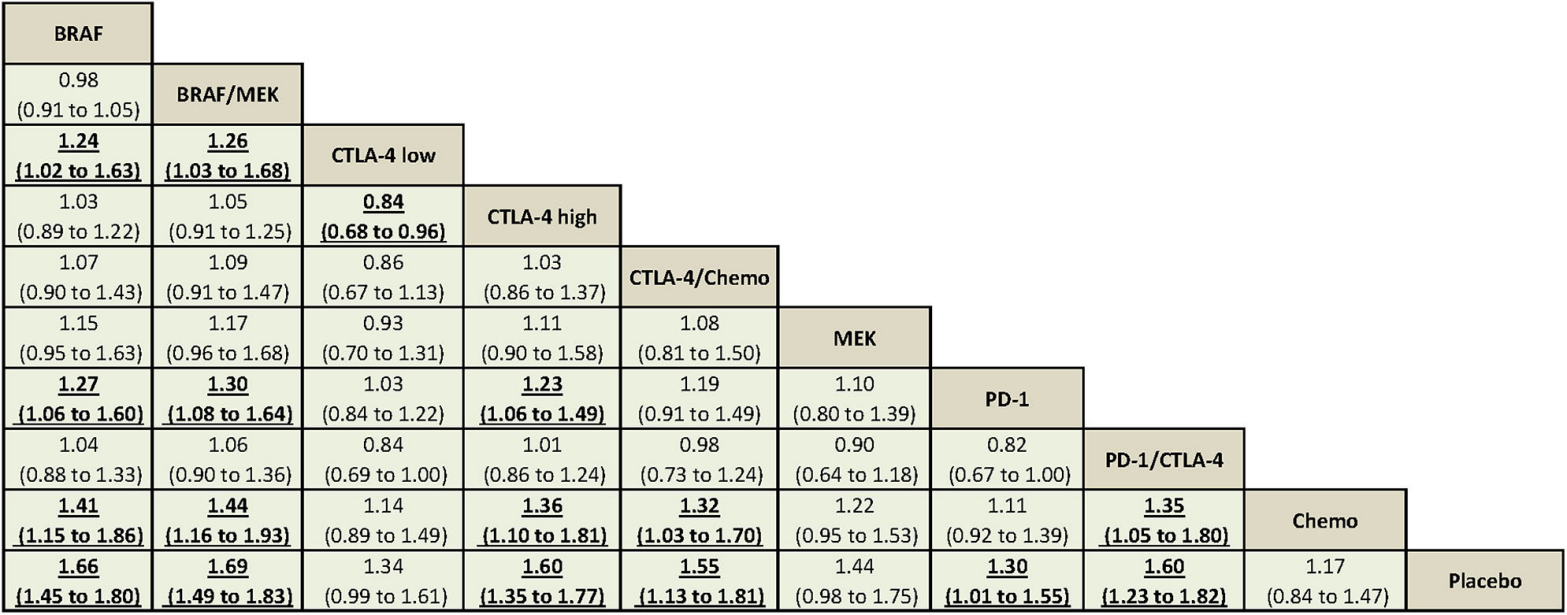
The Bayesian network meta-analysis of overall high-grade AEs. Comparisons should be read from the top treatment to the bottom treatment. Bold underline cells are significant. Results represent the pooled relative risks and 95% credible intervals for overall high-grade AEs. Relative risk >1 favors the bottom treatment.

Using CTLA-4 at a low dose (i.e., ipilimumab at 3 mg/kg) was associated with decreased overall high-grade AEs compared with using CTLA-4 at a high dose (i.e., ipilimumab at 10 mg/kg) (RR = 0.84, 95% CrI = 0.68–0.96). Compared with using PD-1 inhibitor alone, BRAF/MEK, BRAF, and CTLA-4 at a high dose were associated with significantly increased overall high-grade AEs (Figure 2).

### General Symptomatic High-Grade AEs

Twenty-four RCTs (*n* = 12,069) were involved in the NMA of high-grade fatigue (Supplementary Figure 2A). The incidence of fatigue was highest for CTLA-4/chemo (incidence = 0.94%, 95% CrI = 0.78–0.99%, SUCRA = 11.2%), followed by BRAF/MEK (incidence = 0.93%, 95% CrI = 0.82–0.98%, SUCRA = 13.0%) and using BRAF alone (incidence = 0.91%, 95% CrI = 0.77–0.98%, SUCRA = 25.6%). CTLA-4/chemo increased high-grade fatigue significantly compared with PD-1 inhibitor (RR = 1.61, 95% CrI = 1.19–2.74). PD-1 inhibitor was not associated with increased high-grade fatigue compared with placebo (RR = 1.15, 95% CrI = 0.63–1.60) (Supplementary Figure 3A).

Twenty-three RCTs (*n* = 11,927) were involved in the NMA of high-grade pyrexia (Supplementary Figure 2B). The incidence was highest for PD-1/CTLA-4 (incidence = 1.14%, 95% CrI = 0.86–1.19%, SUCRA = 13.5%), followed by high-dose CTLA-4 (incidence = 1.14%, 95% CrI = 0.92–1.19%, SUCRA = 15.9%) and BRAF/MEK (incidence = 1.13%, 95% CrI = 0.95–1.18%, SUCRA = 19.8%). Compared with BRAF/MEK, BRAF was associated with decreased high-grade pyrexia (RR = 0.77, 95% CrI = 0.43–0.94). Using PD-1 inhibitor alone decreased high-grade pyrexia significantly compared with PD-1/CTLA-4 (RR = 0.65, 95% CrI = 0.32–0.90) (Supplementary Figure 3B).

### General Laboratory Results–Related High-Grade AEs

Twenty RCTs (*n* = 11,196) were involved in the NMA of high-grade ALT and AST elevation, respectively (Supplementary Figures 4A,B). The incidence of high-grade ALT elevation was highest for CTLA-4/chemo (incidence = 0.60%, 95% CrI = 0.55–0.60%, SUCRA = 6.2%), followed by PD-1/CTLA-4 (incidence = 0.59%, 95% CrI = 0.55–0.60%, SUCRA = 15.1%). The incidence of high-grade AST elevation was highest for CTLA-4/chemo (incidence = 0.59%, 95% CrI = 0.39–0.60%, SUCRA = 14.8%), followed by high-dose CTLA-4 (incidence = 0.59%, 95% CrI = 0.53–0.60%, SUCRA = 15.6%). Compared with using chemotherapy alone, CTLA-4/chemo respectively increased the risks of high-grade ALT and AST elevation slightly (Supplementary Figures 5A,B).

Fourteen RCTs (*n* = 8,133) were involved in the NMA of high-grade hypertension (Supplementary Figure 4C). MEK had the lowest SUCRA value (30.3%) for high-grade hypertension, followed by CTLA-4/chemo (SUCRA = 36.1%) and BRAF/MEK (SUCRA = 37.1%). Compared with chemotherapy, MEK may increase the risk of high-grade hypertension (RR = 1.26, 95% CrI = 1.00–3.45) (Supplementary Figure 5C).

### Musculoskeletal/Pain–Related High-Grade AEs

Twenty RCTs (*n* = 11,059) were involved in the NMA of high-grade arthralgia (Supplementary Figure 6A). The incidence of arthralgia was highest for BRAF (incidence = 0.39%, 95% CrI = 0.34–0.40%, SUCRA = 11.6%), followed by CTLA-4/chemo (incidence = 0.39%, 95% CrI = 0.13–0.40%, SUCRA = 32.2%). Compared with placebo, BRAF and BRAF/MEK increased high-grade arthralgia significantly (Supplementary Figure 7A).

Eleven RCTs (*n* = 5,655) were involved in the NMA of high-grade myalgia (Supplementary Figure 6B). The SUCRA value was lowest for BRAF (16.8%), followed by BRAF/MEK (SUCRA = 34.1%).

### Gastrointestinal High-Grade AEs

Twenty-four RCTs (*n* = 12,069) were involved in the NMA of high-grade diarrhea (Supplementary Figure 8A). The incidence of diarrhea was highest for CTLA-4/chemo (incidence = 1.31%, 95% CrI = 0.86–1.39%, SUCRA = 8.3%), followed by high-dose CTLA-4 (incidence = 1.21%, 95% CrI = 1.02–1.33%, SUCRA = 13.7%) and BRAF/MEK (incidence = 1.18%, 95% CrI = 0.79–1.35%, SUCRA = 23.9%). Compared with PD-1 inhibitors, CTLA-4/chemo was associated with increased high-grade diarrhea (RR = 1.91, 95% CrI = 1.23–3.51). Using CTLA-4 at a low dose was associated with decreased high-grade diarrhea compared with using CTLA-4 alone at a high dose (RR = 0.85, 95% CrI = 0.61–0.98). Compared with BRAF/MEK, using BRAF alone was associated with decreased high-grade diarrhea (RR = 0.80, 95% CrI = 0.53–0.98) (Supplementary Figure 9A).

Twenty-four RCTs (*n* = 12,069) were involved in the NMA of high-grade nausea (Supplementary Figure 8B). The incidence of nausea was highest for MEK (incidence = 0.37%, 95% CrI = 0.22–0.40%, SUCRA = 23.9%), followed by PD-1/CTLA-4 (SUCRA = 24.4%), BRAF/MEK (SUCRA = 25.9%) and chemotherapy (SUCRA = 26.7%). Compared with chemotherapy, PD-1 inhibitors may be associated with decreased high-grade nausea (RR = 0.79, 95% CrI = 0.40–0.99) (Supplementary Figure 9B).

### Cutaneous High-Grade AEs

Twenty-three RCTs (*n* = 11,823) were involved in the NMA of high-grade rash (Supplementary Figure 10). The incidence of rash was highest for PD-1/CTLA-4 (incidence = 0.94%, 95% CrI = 0.49–1.00%, SUCRA = 19.0%), followed by BRAF (incidence = 0.88%, 95% CrI = 0.57–0.98%, SUCRA = 27.5%) and MEK (incidence = 0.90%, 95% CrI = 0.21–1.00%, SUCRA = 29.9%). PD-1/CTLA-4, BRAF, and MEK were associated with increased high-grade rash compared with chemotherapy (Supplementary Figure 11).

### Model Fit and Inconsistence Check

The posterior mean values of the residual deviance were 47.0, 33.6, 36.4, 30.0, 30.0, 22.5, 29.2, 16.5, 35.3, 32.7, and 35.1 for overall high-grade AEs, fatigue, pyrexia, ALT elevation, AST elevation, hypertension, arthralgia, myalgia, diarrhea, nausea, and rash, respectively. The model's overall fit was relatively satisfactory. Node splitting analyses did not show inconsistency between direct and indirect results for all the outcomes (Supplementary Table 5).

## Discussion

### Summary of Key Findings

This study fills a crucial knowledge gap regarding the comparative risks of high-grade AEs among the current FDA-approved systemic therapies in advanced melanoma. First, we found that the risk of overall high-grade AEs was highest for the BRAF/MEK inhibitor. Second, there were differences in the spectra of high-grade AEs among BRAF-targeted inhibitor (musculoskeletal toxicities and fatigue), MEK-targeted inhibitor (hypertension and nausea), CTLA-4 inhibitor (diarrhea and ALT/AST elevation), and PD-1/CTLA-4 inhibitors (pyrexia and rash). Third, using PD-1 inhibitor alone had the lowest risks of high-grade AEs for patients with advanced melanoma. Fourth, using CTLA-4 inhibitor alone at a low dose (i.e., ipilimumab at 3 mg/kg) decreased overall high-grade AEs significantly compared with using CTLA-4 inhibitor at a high dose (i.e., ipilimumab at 10 mg/kg).

### Comparison With Other Studies

Our study agreed with the result from Franken et al. (5) that using PD-1 inhibitor alone was associated with the lowest risk of high-grade AEs. Devji et al. (7) showed that BRAF/MEK was associated with lower risk of high-grade AEs compared with using BRAF inhibitor alone despite the result was not significant. On the contrary, we found that BRAF/MEK inhibitors had the highest risk of overall high-grade AEs. The differences between our results and the results from Devji and colleagues' study may contribute to the updated trials included in our study (4, 44, 52, 56, 63). In addition, we focused on the FDA-approved targeted inhibitors and ICIs only. We considered that it would be more clinically relevant and would provide more useful evidence into clinical practice.

Previous studies combined high- and low-dose ipilimumab into one arm in analyses (5, 7). In this study, we classified the ipilimumab into the high-dose (10 mg/kg) and low-dose (3 mg/kg) arms when calculating the comparative risks of SAEs. We found that using CTLA-4 inhibitor alone at a high dose (i.e., ipilimumab at 10 mg/kg) was associated with increased risk of high-grade AEs compared with PD-1 or chemotherapy.

### Strength and Limitations of Study

To our knowledge, this is the first and most comprehensive NMA that investigated high-grade AEs among the FDA-approved ICIs and targeted inhibitors for patients with advanced melanoma. Previous NMAs either focused only on treatment efficacy or provided limited information on high-grade AEs. In addition, we classified the treatments by mechanism of action rather than analyzing the drugs separately. Thus, multiple trials would contribute to the comparison between two treatment categories. The network would be concise. It avoided yielding very sparse networks in analyses because of the current limited number of available trials. Combining different drugs of the same class within a single category may introduce heterogeneity. However, the values of the posterior mean of the residual deviance closely corresponded to the number of data points for the outcomes, indicating satisfactory model's fit.

Four limitations should be noted. First, this study provided evidence only on high-grade AEs with limited types. The tolerability of different treatments was not systematically investigated. In addition, because of limited information provided in each of the included studies and the very low incidence of grade 5 AEs (treatment-related deaths), we combined grades 3–4 AEs and grade 5 AEs. We used grades 3–5 AEs as the main outcome of this study. More clinically meaningful outcomes such as all-grade AEs, treatment-related deaths, or treatment discontinuation due to toxicities should be studied to compare the tolerability of different treatments in the future when more trials provide the detailed information. Further researches could also focus on other common AEs such as loss of weight, altered neurobehavioral responses, or other general laboratory results such as changes in blood or lipid profile. Second, the overall high-grade AEs investigated in this study included both non-immune-related reactions and immune-related reactions. The latter was usually late onset. Current clinical trials of ICIs may not have follow-up interval that is long enough to identify the potential risks. Therefore, the incidence of high-grade AEs of ICIs may be underestimated. Standardized method that specifies the clinical criteria for immune-related AEs would be suggested to be published in the future. Third, commentaries, letters, or trials only in conference abstracts were excluded in this study because of the limited information they provided. Publication bias would be a threat if only full-text articles with published data were extracted. Nevertheless, unpublished data from clinicaltrial.gov were obtained in this study to avoid publication bias. Last but not least, individual patient data (IPD) was not accessed in this study. Despite similar inclusion criteria across the included trials have added our confidence in the ability to estimate comparisons across the network of evidence, we still encourage IPD meta-analysis to be conducted in the future because it would provide more detailed patients' characteristics to identify the potential effect modifiers between the treatment options and the high-grade AEs.

### Clinical and Research Implications

This study has obtained some unique clinical findings. First, we found a very similar overall high-grade AE risk between PD-1/CTLA-4 (i.e., nivolumab 1 mg/kg plus ipilimumab 3 mg/kg) and using ipilimumab at 10 mg/kg alone (RR = 1.00, 95% CrI = 0.86–1.24). However, the spectra of toxicity between them were different. Using ipilimumab at 10 mg/kg had a higher risk of diarrhea and ALT/AST elevation, whereas the combination of nivolumab 1 mg/kg and ipilimumab 3 mg/kg had a higher risk of high-grade pyrexia and rash. Second, the combination of ipilimumab and chemotherapy (CTLA-4/chemo) had the highest risk of high-grade fatigue, ALT/AST elevation, and diarrhea. CTLA-4/chemo was likely to be associated with increased overall high-grade AEs compared with using ipilimumab at 3 mg/kg alone, despite statistical significance was not detected. Third, focusing specifically on ipilimumab, we found that using ipilimumab at 3 mg/kg alone had a higher safety ranking compared with ipilimumab at 10 mg/kg. It is to be noted that ipilimumab at 3 mg/kg decreased high-grade diarrhea risk significantly, compared with using ipilimumab at 10 mg/kg (RR = 0.85, 95% CrI = 0.61–0.98). These findings presented above indicate that it is necessary for clinicians to be fully aware of these high-grade AEs and manage them appropriately according to the diagnosis criteria and treatment guidelines used across related trials. These comparative evidences of high-grade AEs could be used as important references when the clinicians balance against the improvements in clinical efficacy among different FDA-approved systemic therapeutic options and perform shared decision making with patients in advanced melanoma during clinical practice.

Two research implications could be noted. First, current RCTs conducted by pharmaceutical companies were mainly used as evidences to support the new drug application. Most RCTs would have chemotherapy as the control group. Direct evidence compared among targeted inhibitors and ICIs is still lacking. For example, BRAF inhibitors and PD-1 inhibitors have never been directly compared. We encourage more RCTs of real-world study be conducted in the future to focus on head-to-head comparisons among targeted inhibitors and ICIs. Second, the outcomes in this study were defined basing on CTCAE, because previous study showed that data from the analysis of AEs by severity to define serious AEs (SAEs) would be more informative (66). However, the safety data provided to FDA for new drug applications usually include only SAEs, which may not adequately reflect the safety signal. We encourage more data from the analysis of AEs by severity be reported in the future.

## Conclusions

This current systematic review and NMA provides the most comprehensive comparison of high-grade AEs between targeted inhibitors and ICIs for the treatment of advanced melanoma. Our results show that different systemic treatment options have varying high-grade AEs and highlight the important risks of BRAF/MEK, CTLA-4/chemo and PD-1/CTLA-4 in advanced melanoma treatment.

## Data Availability Statement

All datasets generated for this study are included in the article/supplementary material.

## Author Contributions

Y-fH, W-jX, and JD: study conception and paper writing. Y-fH and JD: study design and discussion of the findings. Y-fH and H-yF: data extraction and elaboration. Y-fH, W-jX, and H-yF: data analysis and interpretation. Y-fH, W-jX, H-yF, and JD: all coauthors have read and approved the manuscript in its present form, agreed to be personally accountable for the author's own contributions and to ensure that questions related to the accuracy or integrity of any part of the work, even ones in which the author was not personally involved, are appropriately investigated, resolved, and the resolution documented in the literature. All authors contributed to the article and approved the submitted version.

## Conflict of Interest

The authors declare that the research was conducted in the absence of any commercial or financial relationships that could be construed as a potential conflict of interest.

## Supplementary Material

Supplementary Figure 1 Literature search and selection.

Supplementary Figure 2 Network plot of general symptomatic high-grade AEs. The size of the nodes is proportional to the number of trials that involving the connected treatment (nodes). The width of the lines is proportional to the number of comparisons (beside the line) comparing the connected treatment (nodes). The number of patients randomized to receive the treatment is in parentheses. A total of 26 comparisons were analyzed for high-grade fatigue **(A)**; a total of 25 comparisons were analyzed for high-grade pyrexia **(B)**.

Supplementary Figure 3 The Bayesian network meta-analysis of general symptomatic high-grade AEs. Comparisons should be read from the top treatment to the bottom treatment. Bold underline cells are significant. Results represent the pooled relative risks and 95% credible intervals for high-grade fatigue **(A)** and high-grade pyrexia **(B)**. Relative risk >1 favors the bottom treatment.

Supplementary Figure 4 Network plot of general laboratory results related high-grade AEs. The size of the nodes is proportional to the number of trials that involving the connected treatment (nodes). The width of the lines is proportional to the number of comparisons (beside the line) comparing the connected treatment (nodes). The number of patients randomized to receive the treatment is in parentheses. A total of 22 comparisons were analyzed for high-grade ALT elevation **(A)**; a total of 22 comparisons were analyzed for high-grade AST elevation **(B)**; a total of 16 comparisons were analyzed for high-grade hypertension **(C)**.

Supplementary Figure 5 The Bayesian network meta-analysis of general laboratory results related high-grade AEs. Comparisons should be read from the top treatment to the bottom treatment. Bold underline cells are significant. Results represent the pooled relative risks and 95% credible intervals for high-grade ALT elevation **(A)**, high-grade AST elevation **(B)** and high-grade hypertension **(C)**. Relative risk >1 favors the bottom treatment.

Supplementary Figure 6 Network plot of musculoskeletal/pain related high-grade AEs. The size of the nodes is proportional to the number of trials that involving the connected treatment (nodes). The width of the lines is proportional to the number of comparisons (beside the line) comparing the connected treatment (nodes). The number of patients randomized to receive the treatment is in parentheses. A total of 22 comparisons were analyzed for high-grade arthralgia **(A)**; a total of 13 comparisons were analyzed for high-grade myalgia **(B)**.

Supplementary Figure 7 The Bayesian network meta-analysis of musculoskeletal/pain related high-grade AEs. Comparisons should be read from the top treatment to the bottom treatment. Bold underline cells are significant. Results represent the pooled relative risks and 95% credible intervals for high-grade arthralgia **(A)** and high-grade myalgia **(B)**. Relative risk >1 favors the bottom treatment.

Supplementary Figure 8 Network plot of gastrointestinal high-grade AEs. The size of the nodes is proportional to the number of trials that involving the connected treatment (nodes). The width of the lines is proportional to the number of comparisons (beside the line) comparing the connected treatment (nodes). The number of patients randomized to receive the treatment is in parentheses. A total of 26 comparisons were analyzed for high-grade diarrhea **(A)**; a total of 26 comparisons were analyzed for high-grade nausea **(B)**.

Supplementary Figure 9 The Bayesian network meta-analysis of gastrointestinal high-grade AEs. Comparisons should be read from the top treatment to the bottom treatment. Bold underline cells are significant. Results represent the pooled relative risks and 95% credible intervals for high-grade diarrhea **(A)** and high-grade nausea **(B)**. Relative risk >1 favors the bottom treatment.

Supplementary Figure 10 Network plot of cutaneous high-grade AEs. The size of the nodes is proportional to the number of trials that involving the connected treatment (nodes). The width of the lines is proportional to the number of comparisons (beside the line) comparing the connected treatment (nodes). The number of patients randomized to receive the treatment is in parentheses. A total of 25 comparisons were analyzed for high-grade rash.

Supplementary Figure 11 The Bayesian network meta-analysis of cutaneous high-grade AEs. Comparisons should be read from the top treatment to the bottom treatment. Bold underline cells are significant. Results represent the pooled relative risks and 95% credible intervals for high-grade rash. Relative risk >1 favors the bottom treatment.

Supplementary Table 1 Search strategies.

Supplementary Table 2 High-grade adverse events in the included trials (49 articles including 25 randomized controlled trials).

Supplementary Table 3 Arrangement of treatments into treatment classes.

Supplementary Table 4 Risk of bias summary.

Supplementary Table 5 Nodesplit analysis of network meta-analysis in terms of the outcomes.

## Abbreviations

ALT: alanine aminotransferase
AST: aspartate aminotransferase
CrIs: credible intervals
CTLA-4: cytotoxic T-lymphocyte antigen 4 inhibitors
FDA: Food and Drug Administration
ICIs: immune checkpoint inhibitors
PD-1: programmed cell death 1 inhibitors
RR: relative risk.
