# Supplementary figures and images for: Comparative Risks of High-Grade Adverse Events Among FDA-Approved Systemic Therapies in Advanced Melanoma: Systematic Review and Network Meta-Analysis
Source: Front Oncol. 2020 Oct 15;10:571135. doi: 10.3389/fonc.2020.571135 (PMC7593404; doi:10.3389/fonc.2020.571135)

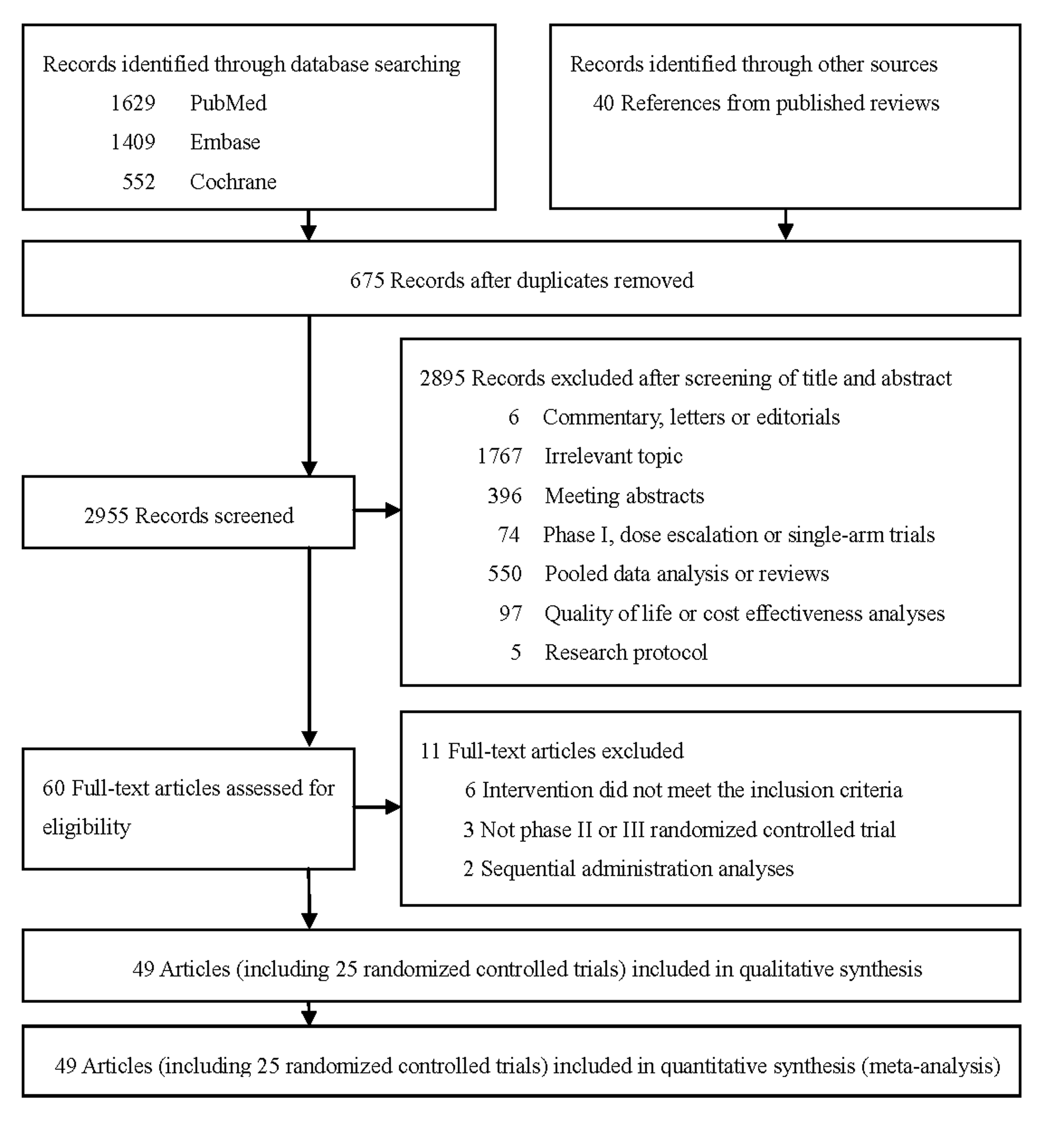

Supplement: Supplementary Figure 1 — Literature search and selection. [file Image_1.TIFF]

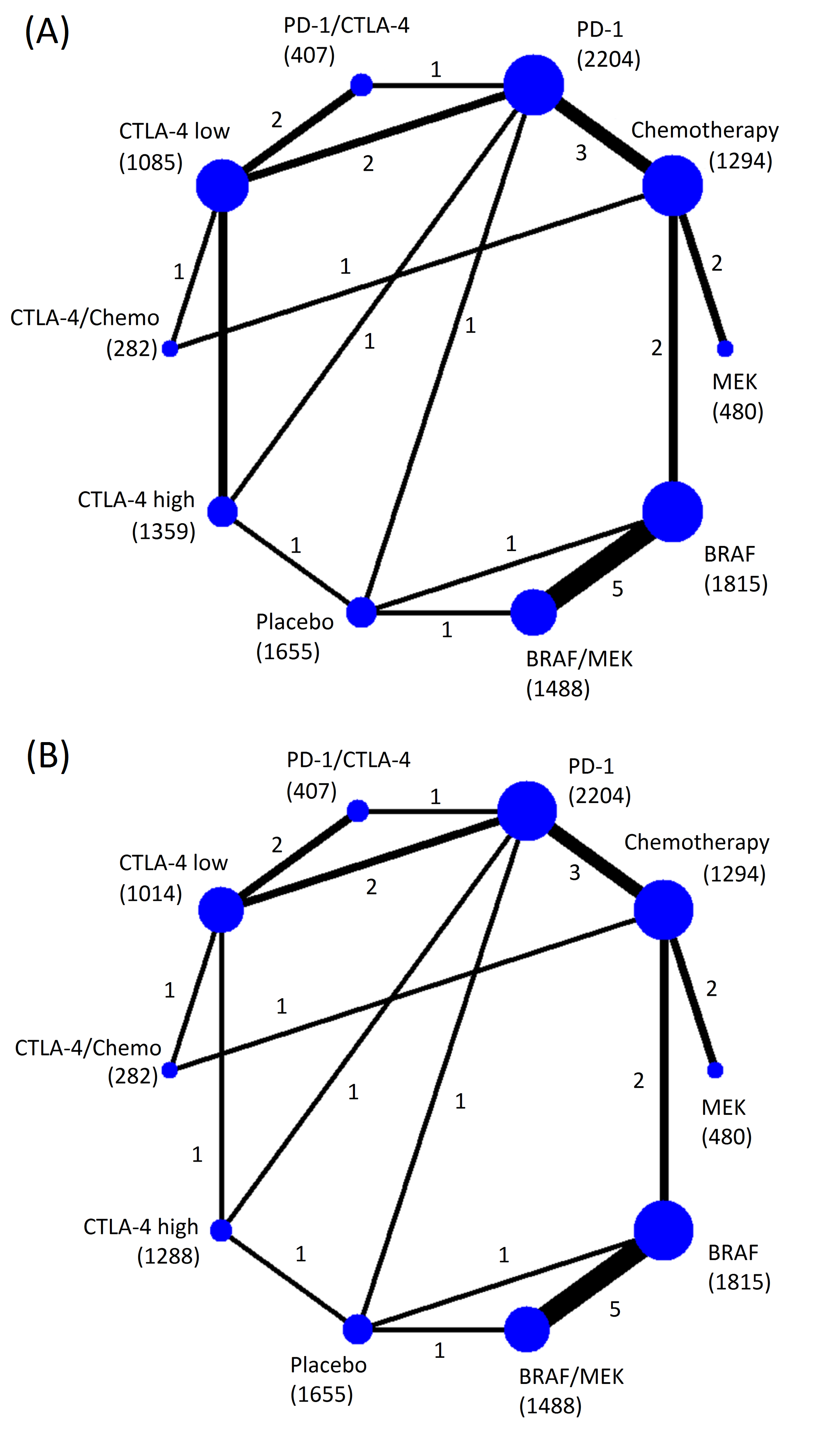

Supplement: Supplementary Figure 2 — Network plot of general symptomatic high-grade AEs. The size of the nodes is proportional to the number of trials that involving the connected treatment (nodes). The width of the lines is proportional to the number of comparisons (beside the line) comparing the connected treatment (nodes). The number of patients randomized to receive the treatment is in parentheses. A total of 26 comparisons were analyzed for high-grade fatigue (A); a total of 25 comparisons were analyzed for high-grade pyrexia (B). [file Image_2.TIF]

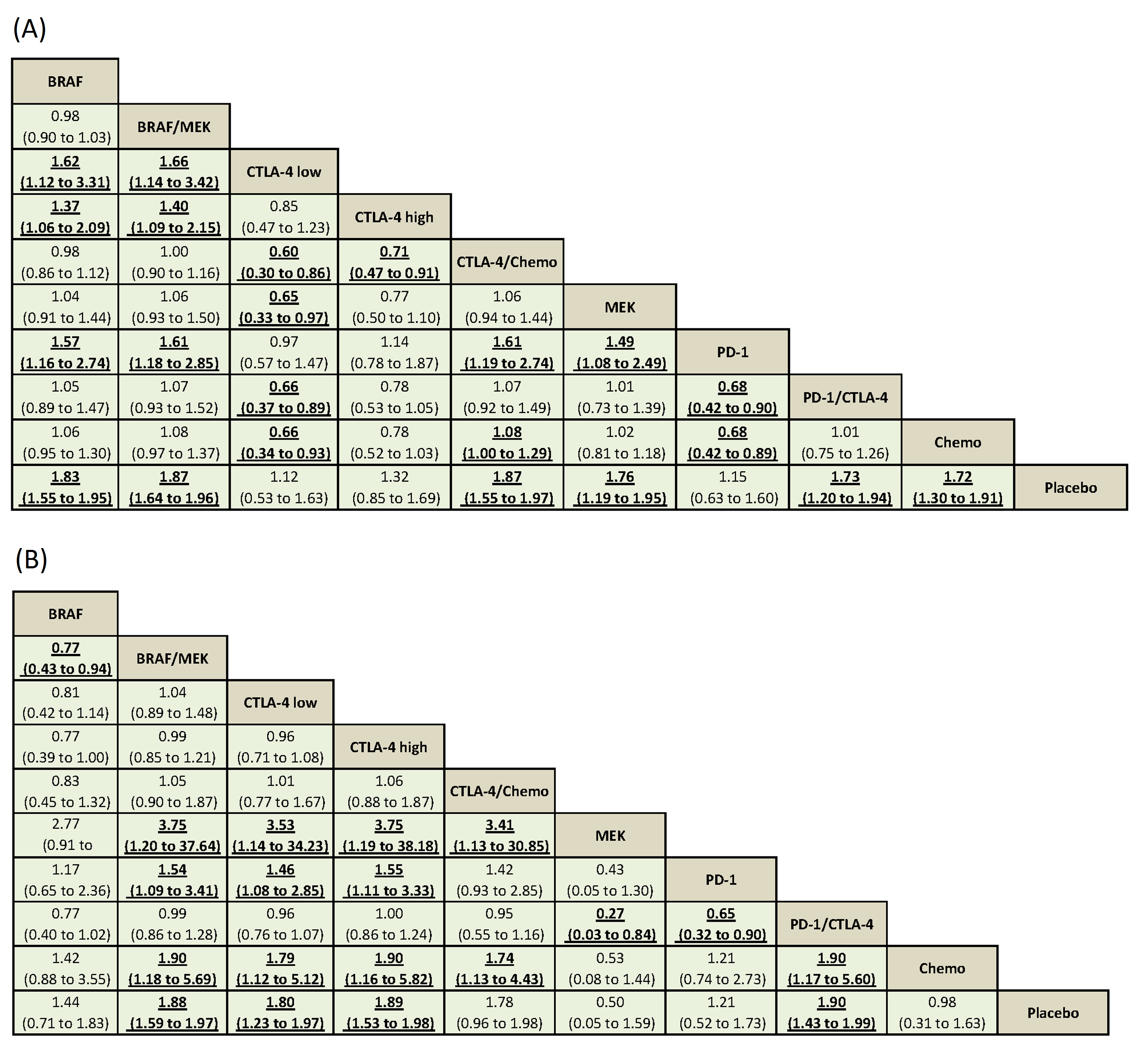

Supplement: Supplementary Figure 3 — The Bayesian network meta-analysis of general symptomatic high-grade AEs. Comparisons should be read from the top treatment to the bottom treatment. Bold underline cells are significant. Results represent the pooled relative risks and 95% credible intervals for high-grade fatigue (A) and high-grade pyrexia (B). Relative risk >1 favors the bottom treatment. [file Image_3.TIFF]

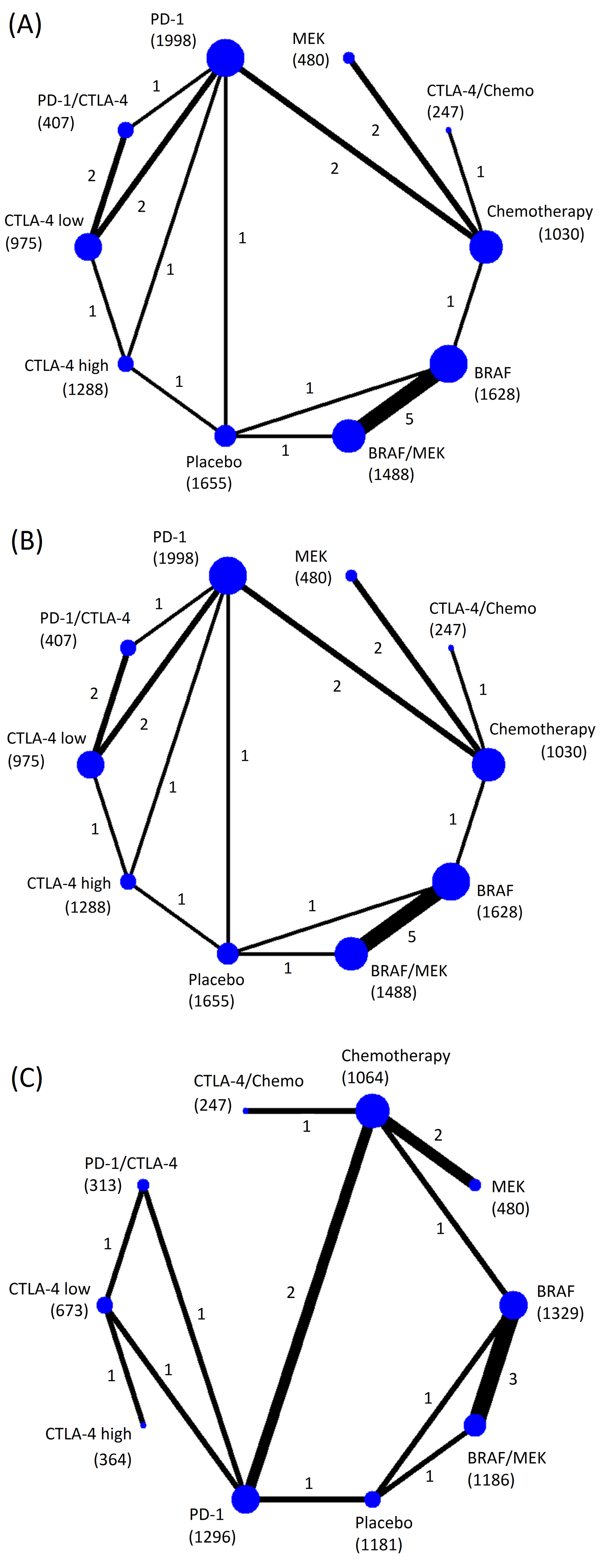

Supplement: Supplementary Figure 4 — Network plot of general laboratory results related high-grade AEs. The size of the nodes is proportional to the number of trials that involving the connected treatment (nodes). The width of the lines is proportional to the number of comparisons (beside the line) comparing the connected treatment (nodes). The number of patients randomized to receive the treatment is in parentheses. A total of 22 comparisons were analyzed for high-grade ALT elevation (A); a total of 22 comparisons were analyzed for high-grade AST elevation (B); a total of 16 comparisons were analyzed for high-grade hypertension (C). [file Image_4.TIF]

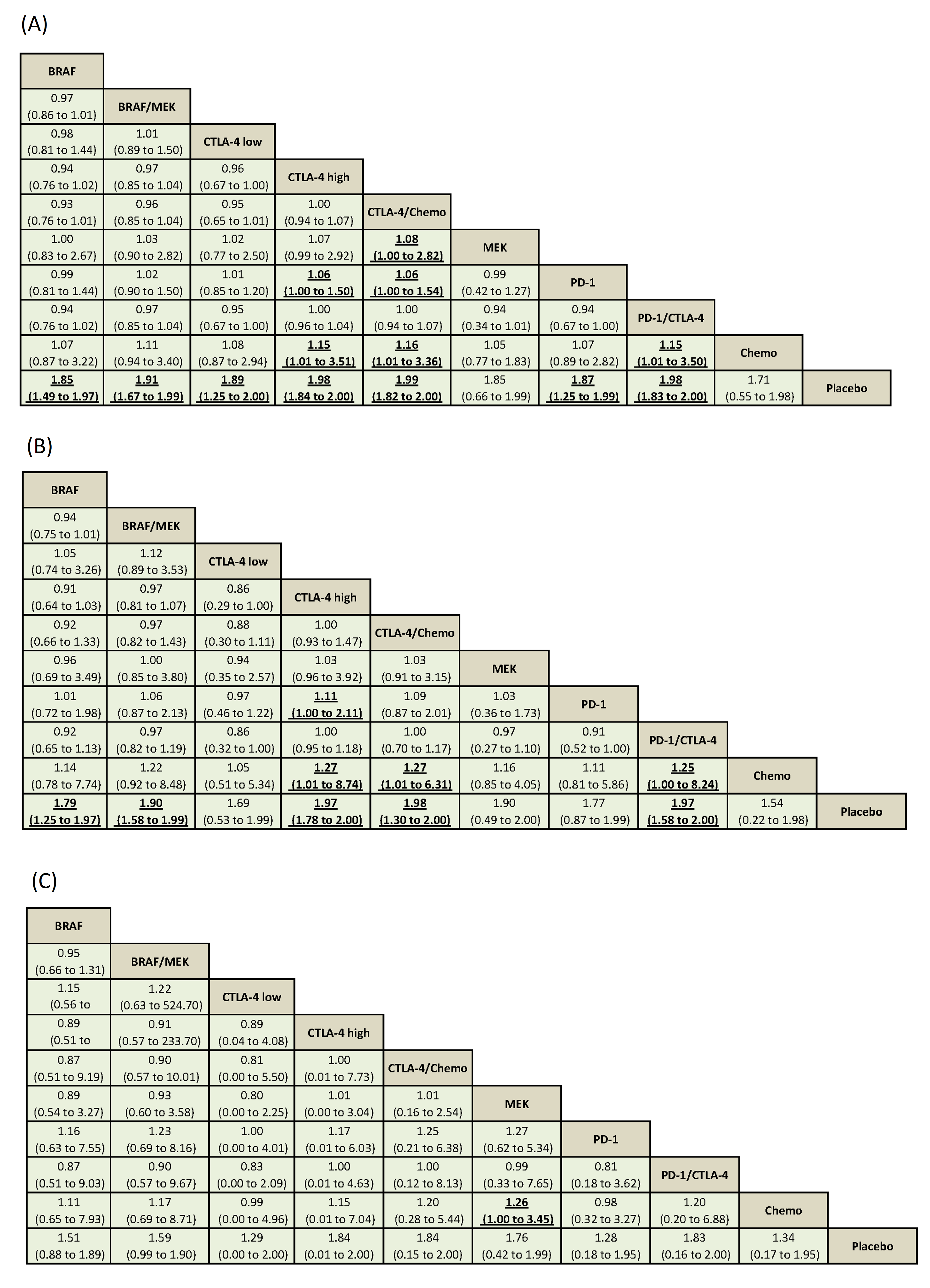

Supplement: Supplementary Figure 5 — The Bayesian network meta-analysis of general laboratory results related high-grade AEs. Comparisons should be read from the top treatment to the bottom treatment. Bold underline cells are significant. Results represent the pooled relative risks and 95% credible intervals for high-grade ALT elevation (A), high-grade AST elevation (B) and high-grade hypertension (C). Relative risk >1 favors the bottom treatment. [file Image_5.TIFF]

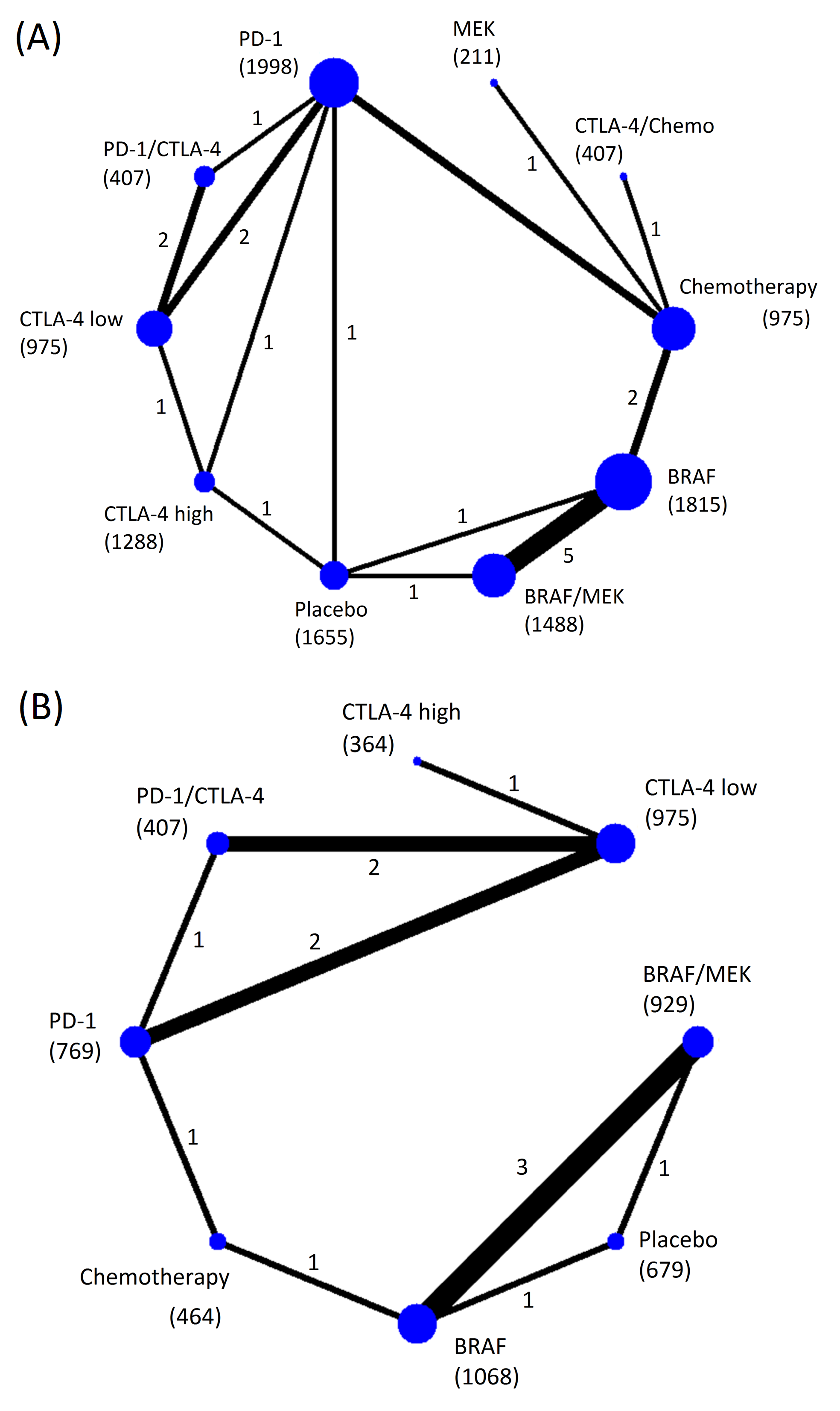

Supplement: Supplementary Figure 6 — Network plot of musculoskeletal/pain related high-grade AEs. The size of the nodes is proportional to the number of trials that involving the connected treatment (nodes). The width of the lines is proportional to the number of comparisons (beside the line) comparing the connected treatment (nodes). The number of patients randomized to receive the treatment is in parentheses. A total of 22 comparisons were analyzed for high-grade arthralgia (A); a total of 13 comparisons were analyzed for high-grade myalgia (B). [file Image_6.TIF]

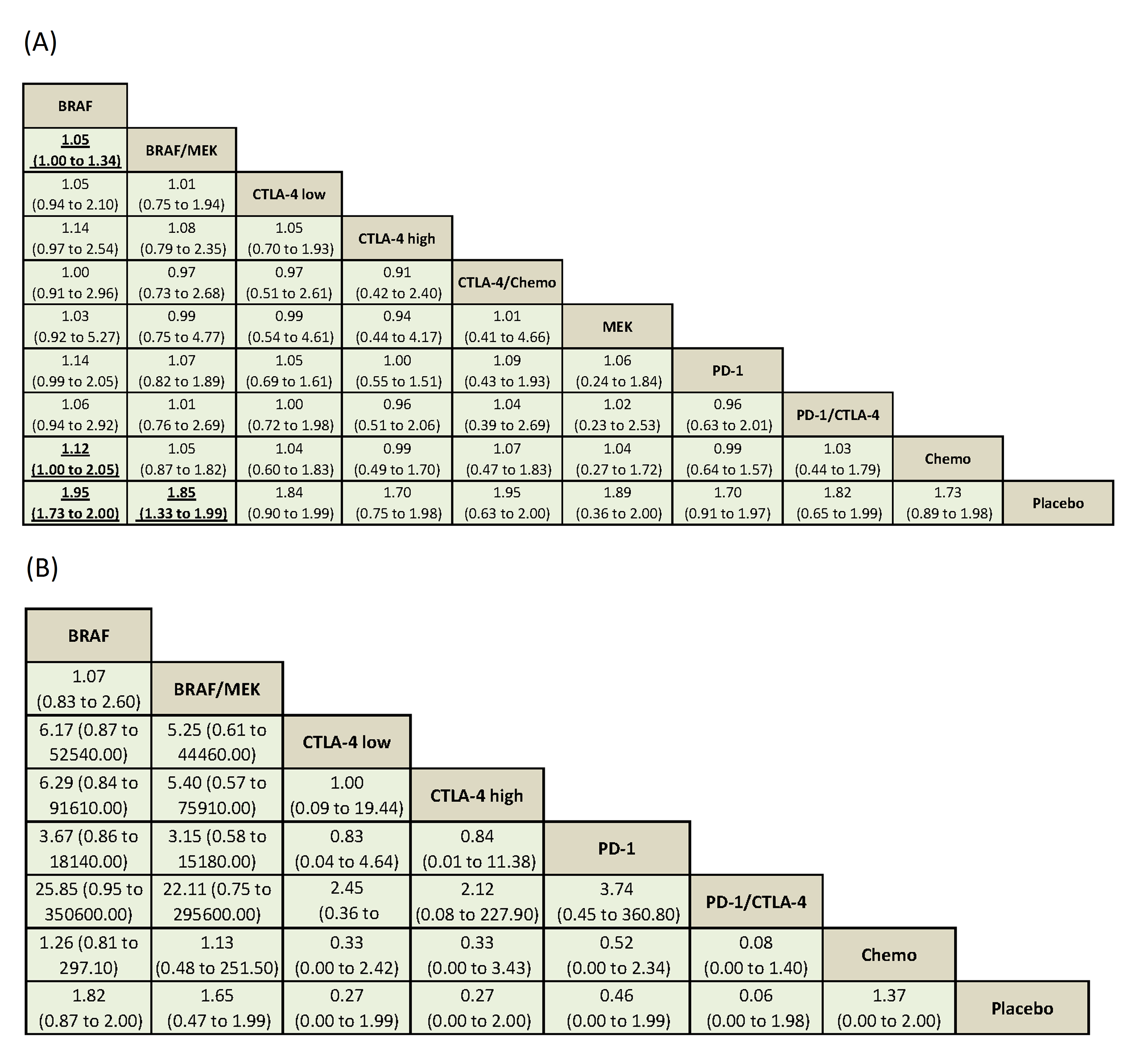

Supplement: Supplementary Figure 7 — The Bayesian network meta-analysis of musculoskeletal/pain related high-grade AEs. Comparisons should be read from the top treatment to the bottom treatment. Bold underline cells are significant. Results represent the pooled relative risks and 95% credible intervals for high-grade arthralgia (A) and high-grade myalgia (B). Relative risk >1 favors the bottom treatment. [file Image_7.TIFF]

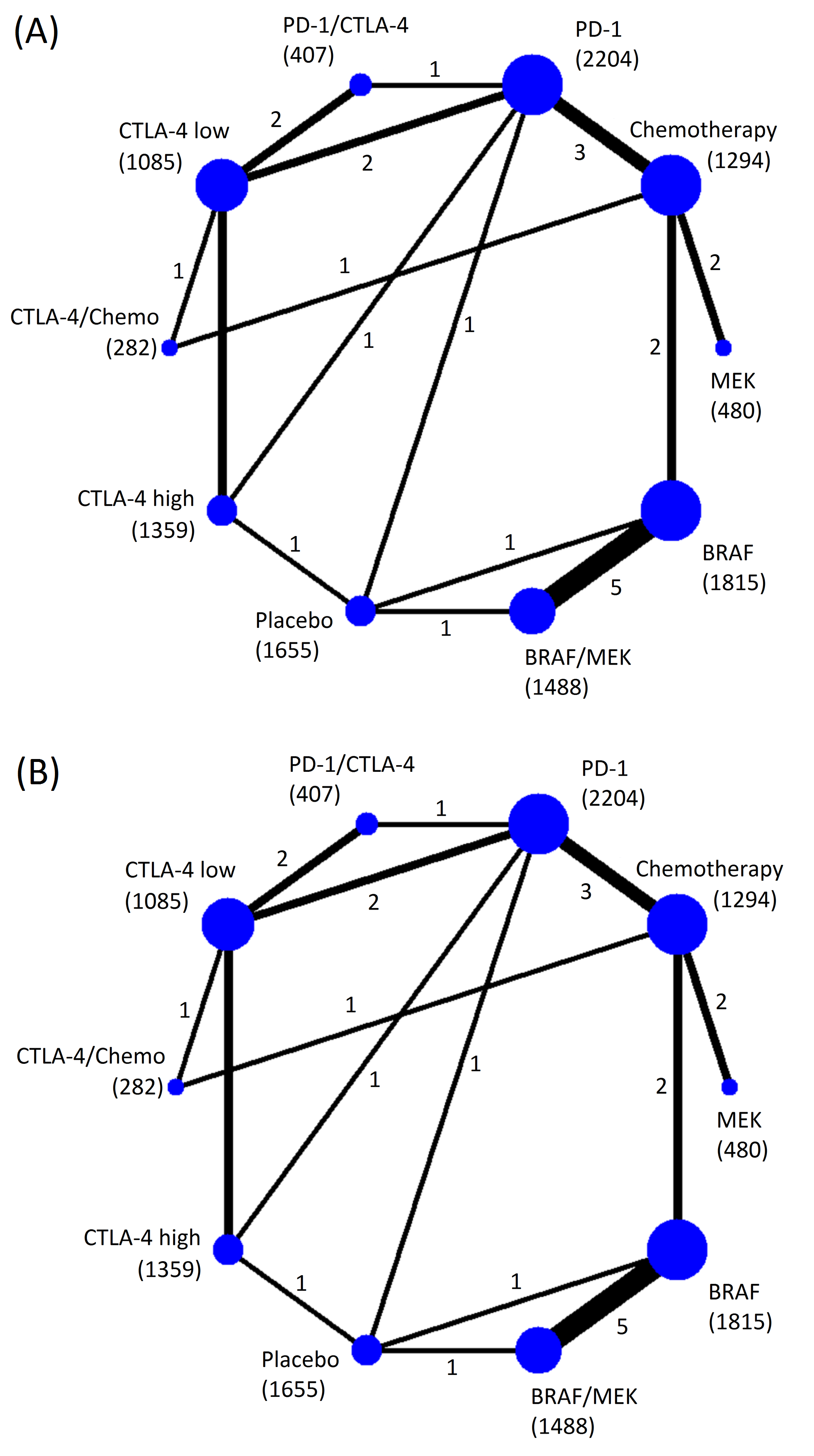

Supplement: Supplementary Figure 8 — Network plot of gastrointestinal high-grade AEs. The size of the nodes is proportional to the number of trials that involving the connected treatment (nodes). The width of the lines is proportional to the number of comparisons (beside the line) comparing the connected treatment (nodes). The number of patients randomized to receive the treatment is in parentheses. A total of 26 comparisons were analyzed for high-grade diarrhea (A); a total of 26 comparisons were analyzed for high-grade nausea (B). [file Image_8.TIF]

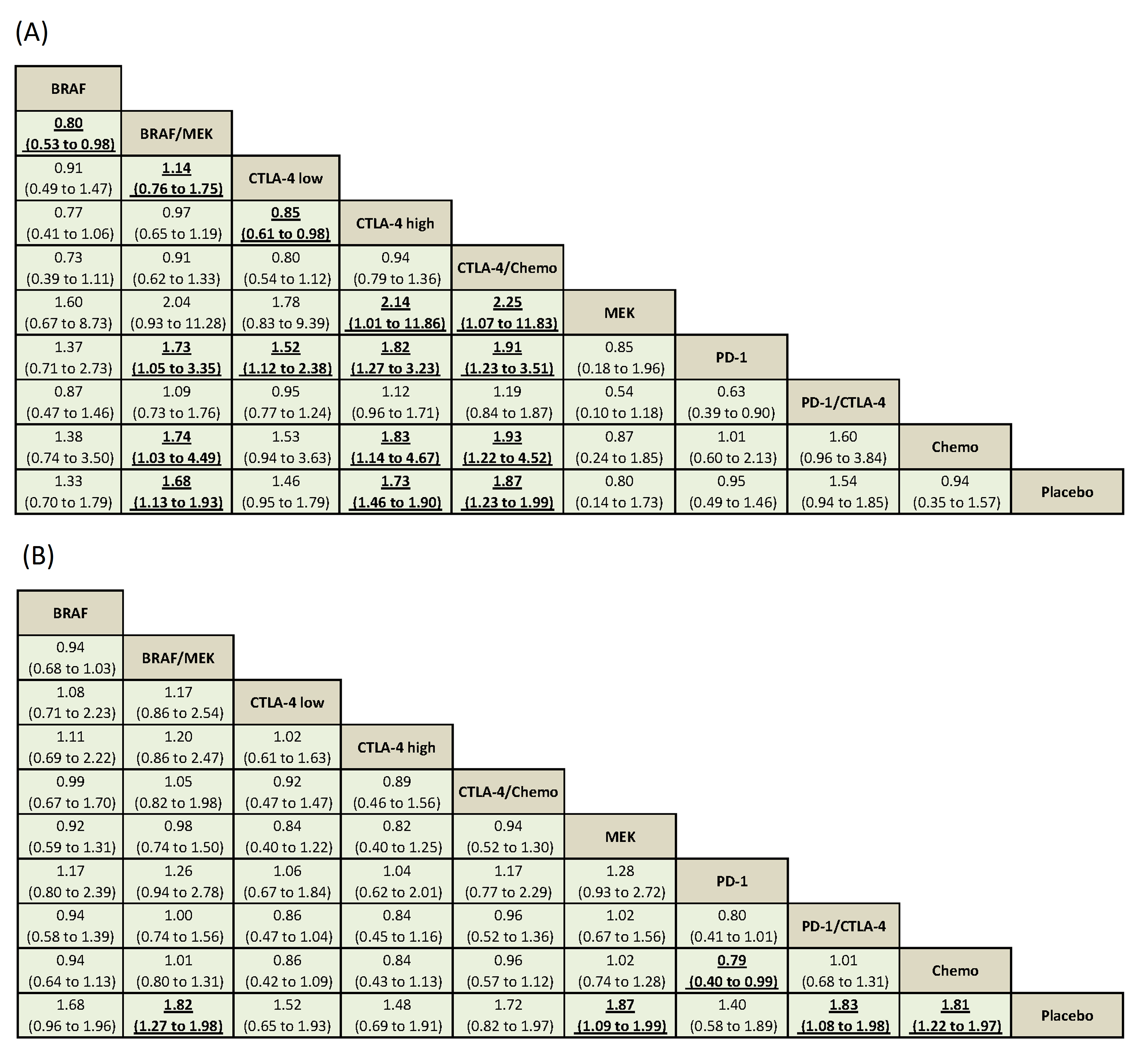

Supplement: Supplementary Figure 9 — The Bayesian network meta-analysis of gastrointestinal high-grade AEs. Comparisons should be read from the top treatment to the bottom treatment. Bold underline cells are significant. Results represent the pooled relative risks and 95% credible intervals for high-grade diarrhea (A) and high-grade nausea (B). Relative risk >1 favors the bottom treatment. [file Image_9.TIFF]

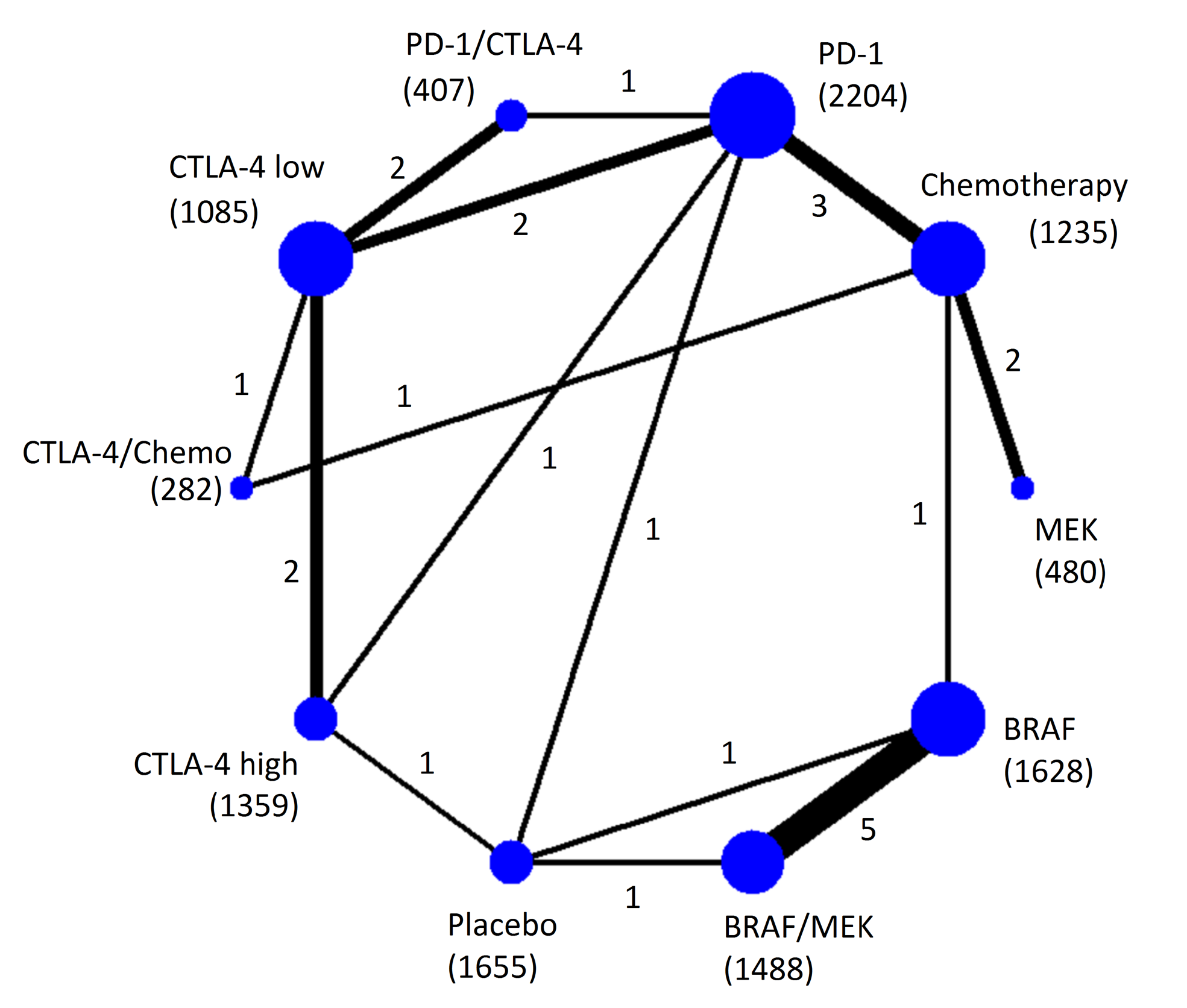

Supplement: Supplementary Figure 10 — Network plot of cutaneous high-grade AEs. The size of the nodes is proportional to the number of trials that involving the connected treatment (nodes). The width of the lines is proportional to the number of comparisons (beside the line) comparing the connected treatment (nodes). The number of patients randomized to receive the treatment is in parentheses. A total of 25 comparisons were analyzed for high-grade rash. [file Image_10.TIF]

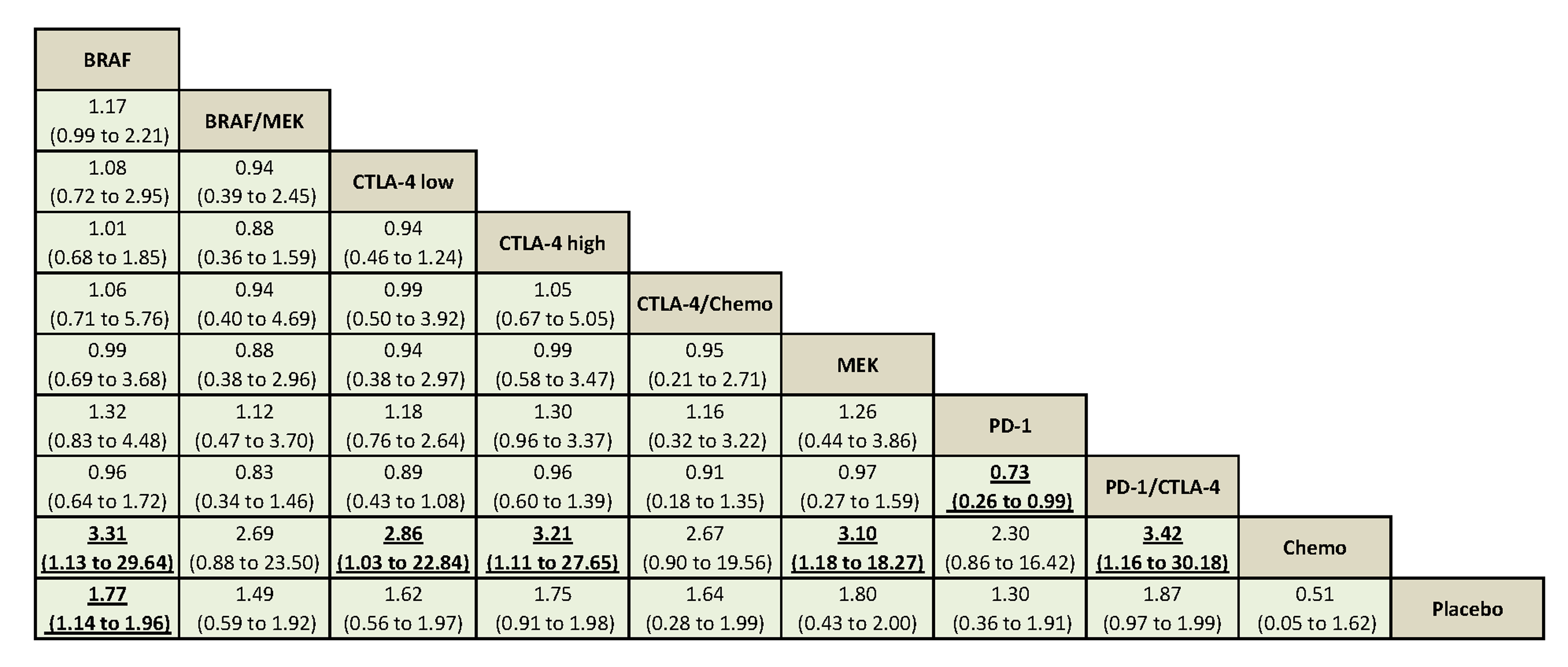

Supplement: Supplementary Figure 11 — The Bayesian network meta-analysis of cutaneous high-grade AEs. Comparisons should be read from the top treatment to the bottom treatment. Bold underline cells are significant. Results represent the pooled relative risks and 95% credible intervals for high-grade rash. Relative risk >1 favors the bottom treatment. [file Image_11.TIFF]
