# Supplementary material for: Comparative Risks of High-Grade Adverse Events Among FDA-Approved Systemic Therapies in Advanced Melanoma: Systematic Review and Network Meta-Analysis
Source: Front Oncol. 2020 Oct 15;10:571135. doi: 10.3389/fonc.2020.571135 (PMC7593404; doi:10.3389/fonc.2020.571135)
Supplement: Supplementary Table 1 — Search strategies. [file Table_1.DOCX]

**Supplementary Table 1 Search strategies**

**PubMed**

| **#** | **Term** | **Results** |
| --- | --- | --- |
| 1 | (melanoma [Title/Abstract] OR melanocyte [Title/Abstract]) AND (Ipilimumab[Supplementary Concept] OR Ipilimumab[Title/Abstract] OR Yervoy[Title/Abstract] OR nivolumab[Supplementary Concept] OR nivolumab[Title/Abstract] OR Opdivo[Title/Abstract] OR pembrolizumab[Supplementary Concept] OR pembrolizumab [Title/Abstract] OR keytruda[Title/Abstract] OR binimetinib [Supplementary Concept] OR binimetinib [Title/Abstract] OR Mektovi [Title/Abstract] OR cobimetinib [Supplementary Concept] OR cobimetinib [Title/Abstract] OR Cotellic [Title/Abstract] OR dabrafenib [Supplementary Concept] OR dabrafenib [Title/Abstract] OR Tafinlar [Title/Abstract] OR encorafenib [Supplementary Concept] OR encorafenib [Title/Abstract] OR Braftovi [Title/Abstract] OR trametinib [Supplementary Concept] OR trametinib [Title/Abstract] OR Mekinist [Title/Abstract] OR vemurafenib [Supplementary Concept] OR vemurafenib [Title/Abstract] OR Zelboraf [Title/Abstract] OR Anti CTLA 4[Title/Abstract] OR Anti-CTLA-4[Title/Abstract] OR cytotoxic T-lymphocyte antigen 4 [Title/Abstract] OR PD 1 [Title/Abstract] OR PD-1 [Title/Abstract] OR programmed cell death 1 receptor [Title/Abstract] OR BRAF [Title/Abstract] OR MEK [Title/Abstract]) AND (random* [Title/Abstract] OR contro* [Title/Abstract] OR placebo [Title/Abstract] OR phase III [Title/Abstract] OR phase II [Title/Abstract] OR phase II/III [Title/Abstract]) | 1629 |

**Embase**

| **#** | **Term** | **Results** |
| --- | --- | --- |
| 1 | 'melanoma'/exp | 203266 |
| 2 | 'melanoma' OR 'melanocyt$' OR 'keratinocyt$':ti,ab,kw | 240271 |
| 3 | #1 OR #2 | 240625 |
| 4 | 'ipilimumab'/exp OR 'Yervoy'/exp OR 'nivolumab'/exp OR 'Opdivo'/exp OR 'pembrolizumab'/exp OR 'Keytruda'/exp OR 'binimetinib'/exp OR 'Mektovi'/exp OR 'cobimetinib'/exp OR 'Cotellic'/exp OR 'dabrafenib'/exp OR 'Tafinlar'/exp OR 'encorafenib'/exp OR 'Braftovi'/exp OR 'trametinib'/exp OR 'Mekinist'/exp OR 'vemurafenib'/exp OR 'Zelboraf'/exp OR 'CTLA 4'/exp OR 'cytotoxic T-lymphocyte antigen 4'/exp OR 'pd1'/exp OR 'programmed cell death 1 receptor'/exp OR 'B Raf kinase inhibitor'/exp OR 'mitogen activated protein kinase inhibitor'/exp OR (ipilimumab OR YervoyOR nivolumab OR Opdivo OR Pembrolizumab OR Keytruda OR binimetinib OR Mektovi OR cobimetinib OR Cotellic OR dabrafenib OR Tafinlar OR encorafenib OR Braftovi OR trametinib OR Mekinist OR vemurafenib OR Zelboraf):ab,ti,kw | 87422 |
| 5 | 'randomized controlled trial'/exp OR 'randomized controlled trial':ti,ab,kw OR 'randomized controlled trials'/exp OR 'randomized controlled trials':ti,ab,kw | 791867 |
| 6 | 'editorial'/de OR 'letter'/de OR 'case report'/de | 3949403 |
| 7 | [animals]/lim NOT [humans]/lim | 5720800 |
| 8 | #6 OR #7 | 9604836 |
| 9 | #5 NOT #8 | 750137 |
| 10 | #3 AND #4 AND #9 | 1409 |

**Cochrane search strategy**

| **#** | **Term** | **Results** |
| --- | --- | --- |
| 1 | melanoma:ti,ab AND (ipilimumab:ti,ab,kw OR nivolumab:ti,ab,kw OR pembrolizumab:ti,ab,kw OR binimetinib:ti,ab,kw OR cobimetinib:ti,ab,kw OR dabrafenib:ti,ab,kw OR encorafenib:ti,ab,kw OR trametinib:ti,ab,kw OR vemurafenib:ti,ab,kw) AND trial:ti,ab NOT review:ti,kw | 552 |
