# Supplementary material for: Comparative Risks of High-Grade Adverse Events Among FDA-Approved Systemic Therapies in Advanced Melanoma: Systematic Review and Network Meta-Analysis
Source: Front Oncol. 2020 Oct 15;10:571135. doi: 10.3389/fonc.2020.571135 (PMC7593404; doi:10.3389/fonc.2020.571135)
Supplement: Supplementary Table 2 — High-grade adverse events in the included trials (49 articles including 25 randomized controlled trials). [file Table_2.docx]

**Supplementary Table 2 High-grade adverse events in the included trials (49 articles including 25 randomized controlled trials)**

| **Trial name** | **Treatment class** | **General, symptomatic high-grade AEs** | | **General, laboratory results related high-grade AEs** | | | **Musculoskeletal/Pain related high-grade AEs** | | **Gastrointestinal high-grade AEs** | | **Cutaneoushigh-grade AEs** |
| --- | --- | --- | --- | --- | --- | --- | --- | --- | --- | --- | --- |
|  |  | Fatigue | Pyrexia | ALT | AST | Hypertension | Arthralgia | Myalgia | Diarrhea | Nausea | Rash |
| BREAK-3 | BRAF | 2 | 10* | NA | NA | NA | 1 | NA | 1* | 1* | NA |
|  | Chemotherapy | 0 | 0 | NA | NA | NA | 0 | NA | 0* | 1* | NA |
| BRF113220 | BRAF/MEK | 2 | 16* | 0* | 0* | NA | 0 | 0 | 1 | 2 | 0 |
|  | BRAF | 4 | 1* | 0* | 0* | NA | 0 | 1 | 0 | 0 | 0 |
| BRIM-3 | BRAF | 10 | 5* | 1* | 0* | 1* | 20 | 1* | 2 | 4 | 28 |
|  | Chemotherapy | 6 | 4* | 1* | 0* | 0* | 3 | 0* | 1 | 5 | 0 |
| BRIM-8 | BRAF | 7 | 0 | 14 | 8 | 6 | 17 | 3 | 5 | 1 | 14 |
|  | Placebo | 1 | 0 | 1 | 1 | 2 | 0 | 0 | 2 | 0 | 3 |
| CA184-004 | CTLA-4 low dose | NA | NA | NA | NA | NA | NA | NA | NA | NA | NA |
|  | CTLA-4 high dose | NA | NA | NA | NA | NA | NA | NA | NA | NA | NA |
| CA184-022 | CTLA-4 low dose | 1 | NA | NA | NA | NA | NA | NA | 1 | 0 | 1 |
|  | CTLA-4 high dose | 2 | NA | NA | NA | NA | NA | NA | 10 | 1 | 0 |
| CA184-024 | CTLA-4 plus chemotherapy | 27 | 19* | 54 | 48* | 1* | 1* | NA | 16* | 4 | 3 |
|  | Chemotherapy | 12 | 4* | 2 | 3 | 0* | 0* | NA | 1* | 7* | 0 |
| CA184-169 | CTLA-4 high dose | 5* | 9* | 12 | 8 | 1 | 0 | 1* | 42* | 2* | 5 |
|  | CTLA-4 low dose | 3* | 5* | 2 | 1 | 0 | 2 | 1 | 22* | 3* | 2 |
| CheckMate 037 | PD-1 | 3 | 4* | 3 | 2 | 1* | 1 | NA | 2* | 2* | 1 |
|  | Chemotherapy | 4 | 2* | 0 | 0 | 0* | 1 | NA | 2* | 2 | 0 |
| CheckMate 066 | PD-1 | 0 | 4* | NA | NA | 0* | NA | NA | 2 | 0* | 1 |
|  | Chemotherapy | 2 | 1* | NA | NA | 1* | NA | NA | 1 | 1* | 0 |
| CheckMate 067 | PD-1 | 3 | 2* | 4 | 3 | 0* | 1 | 1* | 9 | 2* | 1 |
|  | CTLA-4 plus Anti-PD-1 | 13 | 26* | 27 | 19 | 1* | 2 | 0* | 33* | 9* | 10 |
|  | CTLA-4 low dose | 3 | 10* | 5 | 2 | 0* | 0 | 0* | 25* | 2 | 5 |
| CheckMate 069 | CTLA-4 plus Anti-PD-1 | 5 | 6* | 10 | 7 | NA | 0* | 0 | 9 | 1 | 4 |
|  | CTLA-4 low dose | 0 | 3* | 0 | 0 | NA | 1* | 1* | 5 | 1 | 0 |
| CheckMate 238 | PD-1 | 2 | 0 | 5 | 2 | NA | 1 | NA | 7 | 1 | 5 |
|  | CTLA-4 high dose | 4 | 2 | 26 | 19 | NA | 2 | NA | 43 | 0 | 14 |
| coBRIM | BRAF/MEK | 11 | 6* | 28 | 22 | NA | 6 | 1* | 16 | 3 | 42 |
|  | BRAF | 7 | 3* | 15 | 5 | NA | 12 | 1* | 2 | 2 | 40 |
| COLUMBUS | BRAF/MEK | 4 | 7 | 10 | 4 | 12 | 2 | 0 | 4 | 4 | 3 |
|  | BRAF | 4 | 0 | 3 | 3 | 6 | 11 | 1 | 4 | 3 | 6 |
| COMBI-AD | BRAF/MEK | 19 | 67* | 16 | 16 | 25 | 4 | 1 | 4 | 4 | 1* |
|  | Placebo | 1 | 4* | 1 | 1 | 8 | 0 | 0 | 1 | 0 | 1 |
| COMBI-d | BRAF/MEK | 4 | 31* | 4 | 6 | 1* | 1 | NA | 1 | 2* | 0 |
|  | BRAF | 2 | 14* | 1* | 1 | 0* | 0 | NA | 2 | 1 | 1 |
| COMBI-v | BRAF/MEK | 2* | 49* | 5* | 3 | 0* | 3 | NA | 4 | 4* | 4 |
|  | BRAF | 1* | 6* | 8* | 5 | 2* | 15 | NA | 1 | 2 | 30 |
| EORTC 18071 | CTLA-4 high dose | 10 | 18* | 25 | 20 | NA | 2* | NA | 47 | 4* | 6 |
|  | Placebo | 7 | 1* | 0 | 0 | NA | 0* | NA | 10 | 1* | 0 |
| KEYNOTE-002 | PD-1 | 2 | 4* | 1* | 1* | NA | 2* | 0 | 4 | 1 | 0 |
|  | Chemotherapy | 8 | 1* | 0* | 0* | NA | 1 | 1 | 3 | 4 | 0 |
| KEYNOTE-006 | PD-1 | 3 | 0* | 1* | 2* | NA | 0 | 0* | 3 | 0 | 2* |
|  | CTLA-4 low dose | 3 | 4* | 2* | 1* | NA | 0 | 0* | 10* | 1* | 1* |
| KEYNOTE-054 | PD-1 | 4 | 5* | 1* | 1* | 1* | 3 | NA | 4 | 1* | 1 |
|  | Placebo | 2 | 5* | 0* | 0* | 0* | 1* | NA | 3 | 1* | 0 |
| MDX010-08 | CTLA-4 plus chemotherapy | 2 | 1 | NA | NA | NA | NA | NA | 0 | 1 | 1 |
|  | CTLA-4 low dose | 0 | 0 | NA | NA | NA | NA | NA | 0 | 0 | 1 |
| METRIC | MEK | 9 | 3* | 2* | 1* | 32 | 1* | NA | 1 | 2 | 18 |
|  | Chemotherapy | 3 | 4* | 0* | 0* | 6 | 0* | NA | 2 | 1 | 0 |
| NEMO | MEK | 6 | 0 | 7 | 6 | 20 | NA | NA | 4 | 4 | 11 |
|  | Chemotherapy | 3 | 0 | 2 | 0 | 2 | NA | NA | 1 | 1 | 0 |

AEs: adverse events; ALT: alanine aminotransferase; AST: aspartate aminotransferase; CTLA-4: cytotoxic T-lymphocyte-associated antigen-4 inhibitors; NA: not available; PD-1: programmed cell death protein 1 inhibitors.

* Data was extracted from clinicaltrial.gov.
