# Supplementary material for: Comparative Risks of High-Grade Adverse Events Among FDA-Approved Systemic Therapies in Advanced Melanoma: Systematic Review and Network Meta-Analysis
Source: Front Oncol. 2020 Oct 15;10:571135. doi: 10.3389/fonc.2020.571135 (PMC7593404; doi:10.3389/fonc.2020.571135)
Supplement: Supplementary Table 3 — Arrangement of treatments into treatment classes. [file Table_3.DOCX]

**Supplementary Table 3 Arrangement of treatments into treatment classes**

| **Treatment class** | **Treatment** | **Treatment** | **Treatment** | **Treatment** |
| --- | --- | --- | --- | --- |
| BRAF | Dabrafenib 150 mg | Vemurafenib 960 mg | NA | NA |
| BRAF/MEK | Dabrafenib 150 mg plus trametinib 2 mg | Encorafenib 450 mg plus binimetinib 45 mg | Vemurafenib 960 mg plus cobimetinib 60 mg | NA |
| CLTA-4 low dose | Ipilimumab at 3 mg/kg | NA | NA | NA |
| CLTA-4 high dose | Ipilimumab 10 mg/kg | NA | NA | NA |
| CTLA-4/Chemo | Ipilimumab 10 mg/kg plus dacarbazine 850 mg/m² | Ipilimumab 3 mg/kg plus dacarbazine 250mg/m² | NA | NA |
| MEK | Binimetinib 45 mg | Trametinib 2 mg | NA | NA |
| PD-1 | Nivolumab 3 mg/kg | Pembrolizumab 10 mg/kg | Pembrolizumab 200 mg | NA |
| PD-1/CTLA-4 | Nivolumab 1 mg/kg plus ipilimumab 3 mg/kg | NA | NA | NA |
| Chemo | Dacarbazine 1000mg/m² | Carboplatin AUC=6 | Paclitaxel 175mg/m² | Temozolomide (Oral) |
| Placebo | NA | NA | NA | NA |

Chemo: Chemotherapy; CTLA-4: cytotoxic T-lymphocyte-associated antigen-4 inhibitors; NA: not available; PD-1: programmed cell death protein 1 inhibitors.
