# Supplementary material for: Comparative Risks of High-Grade Adverse Events Among FDA-Approved Systemic Therapies in Advanced Melanoma: Systematic Review and Network Meta-Analysis
Source: Front Oncol. 2020 Oct 15;10:571135. doi: 10.3389/fonc.2020.571135 (PMC7593404; doi:10.3389/fonc.2020.571135)
Supplement: Supplementary Table 4 — Risk of bias summary. [file Table_4.DOCX]

**Supplementary Table 4 Risk of bias summary**

| **Trial** | **Random sequence generation** | **Allocation concealment** | **Blinding of participants and personnel** | **Blinding of outcome assessment** | **Incomplete outcome data** | **Selective reporting** | **Other sources of bias** |
| --- | --- | --- | --- | --- | --- | --- | --- |
| BREAK-3 | Low | Low | High | Low | Low | Low | Low |
| BRF113220 | Unclear | Unclear | High | Low | Low | Low | Low |
| BRIM-3 | Low | Low | High | Low | Low | Low | Low |
| BRIM-8 | Low | Low | Low | Low | Low | Low | Low |
| CA184-004 | Unclear | Unclear | Low | Low | Low | High | Low |
| CA184-022 | Low | Low | Low | Low | Low | Low | Low |
| CA184-024 | Low | Low | Low | Low | Low | Low | Low |
| CA184-169 | Low | Low | Low | Low | Low | Low | Low |
| CheckMate 037 | Low | Low | High | Low | Low | Low | Low |
| CheckMate 066 | Low | Low | Low | Low | Low | Low | Low |
| CheckMate 067 | Low | Low | Low | Low | Low | Low | Low |
| CheckMate 069 | Low | Low | Low | Low | Low | Low | Low |
| CheckMate 238 | Low | Low | Low | Low | Low | Low | Low |
| coBRIM | Low | Low | Low | Low | Low | Low | Low |
| COLUMBUS | Low | Low | High | Low | Low | Low | Low |
| COMBI-AD | Unclear | Unclear | Low | Low | Low | Low | Low |
| COMBI-d | Unclear | Unclear | Low | Low | Low | Low | Low |
| COMBI-v | Unclear | Unclear | High | Low | Low | Low | Low |
| EORTC 18071 | Low | Low | Low | Low | Low | Low | Low |
| KEYNOTE-002 | Low | Low | High | Low | Low | Low | Low |
| KEYNOTE-006 | Low | Low | High | Low | Low | Low | Low |
| KEYNOTE-054 | Low | Low | Low | Low | Low | Low | Low |
| MDX010-08 | Low | Low | High | Low | Low | Low | Low |
| METRIC | Unclear | Unclear | High | Low | Low | Low | Low |
| NEMO | Low | Low | High | Low | Low | Low | Low |
