# Supplementary material for: Comparative Risks of High-Grade Adverse Events Among FDA-Approved Systemic Therapies in Advanced Melanoma: Systematic Review and Network Meta-Analysis
Source: Front Oncol. 2020 Oct 15;10:571135. doi: 10.3389/fonc.2020.571135 (PMC7593404; doi:10.3389/fonc.2020.571135)
Supplement: Supplementary Table 5 — Nodesplit analysis of network meta-analysis in terms of the outcomes. [file Table_5.docx]

**Supplementary Table 5 Nodesplit analysis of network meta-analysis in terms of the outcomes**

| **Nodes** | **Direct, RR (95% CI)** | **Indirect, RR (95% CI)** | **Difference, RR (95% CI)** | **P**** |
| --- | --- | --- | --- | --- |
| **Overall high-grade AEs** |  |  |  |  |
| PD-1, Placebo | 1.53 (0.94 to 2.47) | 1.53 (1.00 to 2.33) | 1.00 (0.53 to 1.89) | 0.996 |
| CTLA-4 high, Placebo | 2.11 (1.33 to 3.34) | 2.57 (1.57 to 4.20) | 0.82 (0.42 to 1.61) | 0.568 |
| BRAF/MEK, Placebo | 2.95 (1.79 to 4.85) | 3.45 (2.20 to 5.42) | 0.85 (0.44 to 1.67) | 0.643 |
| BRAF, Placebo | 3.84 (2.28 to 6.46) | 2.71 (1.82 to 4.02) | 1.42 (0.74 to 2.73) | 0.294 |
| PD-1, Chemotherapy | 1.23 (0.92 to 1.64) | 0.91 (0.51 to 1.62) | 1.35 (0.71 to 2.58) | 0.358 |
| MEK, Chemotherapy* | 1.43 (0.99 to 2.07) | 0.50 (NE) | 2.89 (NE) | 0.984 |
| CTLA-4/Chemo, Chemotherapy | 1.43 (0.92 to 2.22) | 2.13 (0.72 to 6.25) | 0.67 (0.21 to 2.15) | 0.502 |
| BRAF, Chemotherapy | 2.17 (1.45 to 3.25) | 2.71 (1.50 to 4.91) | 0.80 (0.39 to 1.64) | 0.541 |
| PD-1, PD-1/CTLA-4 | 0.84 (0.56 to 1.27) | 0.53 (0.29 to 0.97) | 1.58 (0.76 to 3.28) | 0.217 |
| CTLA-4 low, PD-1/CTLA-4* | 0.72 (0.52 to 1.02) | 1.27 (0.48 to 3.36) | 0.57 (0.20 to 1.60) | 0.287 |
| CTLA-4 high, PD-1 | 2.19 (1.51 to 3.17) | 1.22 (0.91 to 1.63) | 1.80 (1.12 to 2.89) | 0.015 |
| CTLA-4 low, PD-1 | 0.98 (0.71 to 1.34) | 1.21 (0.80 to 1.85) | 0.80 (0.47 to 1.36) | 0.418 |
| CTLA-4 low, CTLA-4/Chemo | 0.60 (0.22 to 1.65) | 0.89 (0.50 to 1.58) | 0.67 (0.21 to 2.15) | 0.501 |
| CTLA-4 low, CTLA-4 high | 0.80 (0.60 to 1.07) | 0.52 (0.34 to 0.80) | 1.54 (0.92 to 2.59) | 0.102 |
| BRAF, BRAF/MEK | 0.94 (0.76 to 1.16) | 1.10 (0.58 to 2.09) | 0.85 (0.44 to 1.67) | 0.642 |
| **Fatigue** |  |  |  |  |
| PD-1, Placebo | 1.97 (0.36 to 10.72) | 1.03 (0.31 to 3.47) | 1.92 (0.24 to 15.40) | 0.541 |
| CTLA-4 high, Placebo | 1.44 (0.55 to 3.75) | 3.69 (0.66 to 20.79) | 0.39 (0.05 to 2.81) | 0.349 |
| BRAF/MEK, Placebo | 18.87 (2.54 to 140.33) | 8.30 (1.90 to 36.27) | 2.27 (0.19 to 27.42) | 0.518 |
| BRAF, Placebo | 7.00 (0.87 to 56.48) | 8.95 (2.34 to 34.18) | 0.78 (0.07 to 9.35) | 0.846 |
| PD-1, Chemotherapy | 0.25 (0.09 to 0.69) | 0.22 (0.04 to 1.33) | 1.15 (0.15 to 8.98) | 0.894 |
| MEK, Chemotherapy* | 1.11 (0.44 to 2.83) | 0.04 (NE) | 31.17 (NE) | 0.989 |
| CTLA-4/Chemo, Chemotherapy | 2.29 (1.19 to 4.41) | 1.19 (0.05 to 30.70) | 1.92 (0.07 to 52.91) | 0.7 |
| BRAF, Chemotherapy | 1.47 (0.57 to 3.79) | 2.38 (0.30 to 18.99) | 0.62 (0.06 to 6.06) | 0.679 |
| PD-1, PD-1/CTLA-4 | 0.24 (0.07 to 0.80) | 0.22 (0.02 to 2.77) | 1.06 (0.06 to 20.37) | 0.969 |
| CTLA-4 low, PD-1/CTLA-4* | 0.22 (0.07 to 0.70) | 0.21 (0.01 to 6.10) | 1.07 (0.02 to 46.94) | 0.973 |
| CTLA-4 high, PD-1 | 2.00 (0.37 to 10.84) | 1.19 (0.38 to 3.73) | 1.68 (0.22 to 13.00) | 0.618 |
| CTLA-4 low, PD-1 | 1.04 (0.34 to 3.22) | 0.82 (0.19 to 3.59) | 1.27 (0.20 to 8.11) | 0.801 |
| CTLA-4 low, CTLA-4/Chemo | 0.18 (0.01 to 3.63) | 0.09 (0.02 to 0.38) | 1.92 (0.07 to 52.90) | 0.7 |
| CTLA-4 low, CTLA-4 high | 0.57 (0.17 to 1.95) | 0.91 (0.19 to 4.28) | 0.63 (0.09 to 4.56) | 0.649 |
| BRAF, BRAF/MEK | 0.79 (0.42 to 1.48) | 0.35 (0.03 to 3.88) | 2.27 (0.19 to 27.41) | 0.518 |
| **Pyrexia** |  |  |  |  |
| PD-1, Placebo | 0.99 (0.21 to 4.65) | 2.78 (0.45 to 17.38) | 0.35 (0.03 to 3.91) | 0.397 |
| CTLA-4 high, Placebo | 18.11 (1.90 to 172.55) | 10.81 (1.23 to 94.73) | 1.68 (0.07 to 38.32) | 0.746 |
| BRAF/MEK, Placebo | 16.63 (4.28 to 64.66) | 5.16 (0.62 to 42.54) | 3.23 (0.26 to 39.67) | 0.36 |
| BRAF, Placebo | 1.00 (0.02 to 56.45) | 2.73 (0.75 to 9.99) | 0.37 (0.01 to 25.35) | 0.642 |
| PD-1, Placebo | 1.92 (0.55 to 6.72) | 0.44 (0.06 to 3.45) | 4.39 (0.39 to 48.91) | 0.229 |
| MEK, Chemotherapy* | 0.36 (0.07 to 1.82) | 0.83 (NE) | 0.44 (NE) | 0.998 |
| CTLA-4/Chemo, Chemotherapy | 4.83 (1.13 to 20.67) | 31.31 (0.75 to 1306.54) | 0.15 (0.00 to 8.46) | 0.36 |
| BRAF, Chemotherapy | 1.65 (0.40 to 6.85) | 4.17 (0.41 to 42.44) | 0.39 (0.03 to 6.13) | 0.507 |
| PD-1, PD-1/CTLA-4 | 0.07 (0.01 to 0.35) | 0.24 (0.02 to 2.62) | 0.28 (0.02 to 5.12) | 0.394 |
| CTLA-4 low, PD-1/CTLA-4* | 0.54 (0.20 to 1.49) | 0.27 (0.00 to 34.85) | 1.99 (0.01 to 290.64) | 0.786 |
| CTLA-4 high, PD-1 | 4.99 (0.21 to 121.17) | 10.62 (2.12 to 53.30) | 0.47 (0.01 to 16.77) | 0.679 |
| CTLA-4 low, PD-1 | 5.23 (1.09 to 25.06) | 5.02 (0.73 to 34.41) | 1.04 (0.09 to 11.52) | 0.973 |
| CTLA-4 low, CTLA-4/Chemo | 0.30 (0.01 to 8.30) | 1.95 (0.21 to 18.27) | 0.15 (0.00 to 8.46) | 0.36 |
| CTLA-4 low, CTLA-4 high | 0.56 (0.13 to 2.48) | 0.59 (0.05 to 6.87) | 0.95 (0.05 to 16.95) | 0.974 |
| BRAF, BRAF/MEK | 0.23 (0.12 to 0.47) | 0.07 (0.01 to 0.80) | 3.23 (0.26 to 39.67) | 0.36 |
| **ALT elevation** |  |  |  |  |
| PD-1, Placebo | 2.96 (0.12 to 72.46) | 15.15 (1.49 to 153.52) | 0.20 (0.00 to 10.13) | 0.418 |
| CTLA-4 high, Placebo | 51.32 (3.13 to 840.64) | 47.59 (3.57 to 633.71) | 1.08 (0.02 to 48.72) | 0.969 |
| BRAF/MEK, Placebo | 15.89 (2.12 to 119.29) | 15.72 (2.41 to 102.58) | 1.01 (0.06 to 15.86) | 0.994 |
| BRAF, Placebo | 14.00 (1.86 to 105.65) | 6.68 (1.05 to 42.48) | 2.10 (0.14 to 32.48) | 0.596 |
| PD-1, Chemotherapy | 2.76 (0.32 to 24.19) | 0.41 (0.01 to 18.30) | 6.72 (0.08 to 531.20) | 0.393 |
| MEK, Chemotherapy* | 1.63 (0.41 to 6.52) | 0.03 (NE) | 51.46 (NE) | 0.991 |
| CTLA-4/Chemo, Chemotherapy* | 27.44 (6.76 to 111.31) | 0.01 (NE) | 4974.21 (NE) | 0.962 |
| BRAF, Chemotherapy | 0.87 (0.05 to 13.88) | 5.88 (0.20 to 173.05) | 0.15 (0.00 to 11.74) | 0.392 |
| PD-1, PD-1/CTLA-4 | 0.15 (0.06 to 0.43) | 0.11 (0.01 to 1.64) | 1.43 (0.07 to 29.38) | 0.817 |
| CTLA-4 low, PD-1/CTLA-4* | 0.17 (0.07 to 0.43) | 0.18 (0.01 to 6.23) | 0.98 (0.02 to 44.28) | 0.99 |
| CTLA-4 high, PD-1 | 5.19 (2.01 to 13.39) | 7.66 (1.44 to 40.68) | 0.68 (0.10 to 4.62) | 0.691 |
| CTLA-4 low, PD-1 | 1.43 (0.45 to 4.50) | 0.81 (0.15 to 4.34) | 1.76 (0.23 to 13.34) | 0.584 |
| CTLA-4 low, CTLA-4 high | 0.17 (0.04 to 0.74) | 0.26 (0.06 to 1.11) | 0.65 (0.08 to 5.19) | 0.682 |
| BRAF, BRAF/MEK | 0.59 (0.37 to 0.95) | 0.59 (0.04 to 8.84) | 1.01 (0.06 to 15.86) | 0.994 |
| **AST elevation** |  |  |  |  |
| PD-1, Placebo | 2.96 (0.11 to 78.05) | 7.84 (0.54 to 113.01) | 0.38 (0.01 to 25.72) | 0.651 |
| CTLA-4 high, Placebo | 41.26 (2.29 to 741.97) | 43.58 (2.09 to 910.79) | 0.95 (0.01 to 62.75) | 0.98 |
| BRAF/MEK, Placebo | 15.89 (1.85 to 136.57) | 12.91 (1.46 to 114.01) | 1.23 (0.06 to 26.29) | 0.894 |
| BRAF, Placebo | 8.00 (0.89 to 72.00) | 5.77 (0.72 to 46.24) | 1.39 (0.07 to 28.63) | 0.832 |
| PD-1, Chemotherapy | 2.32 (0.24 to 21.99) | 0.44 (0.00 to 56.29) | 5.23 (0.03 to 1092.98) | 0.544 |
| MEK, Chemotherapy* | 3.00 (0.34 to 26.60) | 0.06 (NE) | 52.25 (NE) | 0.992 |
| CTLA-4/Chemo, Chemotherapy* | 16.26 (4.34 to 60.95) | 0.02 (NE) | 693.15 (NE) | 0.972 |
| BRAF, Chemotherapy | 0.87 (0.02 to 46.47) | 4.58 (0.13 to 162.58) | 0.19 (0.00 to 39.78) | 0.543 |
| PD-1, PD-1/CTLA-4 | 0.16 (0.04 to 0.63) | 0.16 (0.01 to 4.23) | 0.97 (0.03 to 36.58) | 0.987 |
| CTLA-4 low, PD-1/CTLA-4* | 0.11 (0.03 to 0.47) | 0.13 (0.00 to 14.68) | 0.83 (0.00 to 145.02) | 0.944 |
| CTLA-4 high, PD-1 | 9.48 (1.86 to 48.40) | 5.77 (0.64 to 52.29) | 1.64 (0.11 to 25.50) | 0.722 |
| CTLA-4 low, PD-1 | 0.62 (0.13 to 2.85) | 1.15 (0.10 to 13.33) | 0.54 (0.03 to 9.93) | 0.679 |
| CTLA-4 low, CTLA-4 high | 0.13 (0.01 to 1.14) | 0.07 (0.01 to 0.57) | 1.80 (0.09 to 37.78) | 0.706 |
| BRAF, BRAF/MEK | 0.48 (0.22 to 1.05) | 0.39 (0.02 to 7.43) | 1.23 (0.06 to 26.29) | 0.894 |
| **Hypertension** |  |  |  |  |
| PD-1, Placebo | 2.96 (0.12 to 72.46) | 0.52 (0.01 to 29.27) | 5.66 (0.03 to 968.78) | 0.509 |
| BRAF/MEK, Placebo | 3.10 (1.42 to 6.80) | 5.65 (0.98 to 32.62) | 0.55 (0.08 to 3.75) | 0.541 |
| BRAF, Placebo | 3.00 (0.61 to 14.72) | 2.07 (0.66 to 6.51) | 1.45 (0.20 to 10.32) | 0.71 |
| PD-1, Chemotherapy | 0.62 (0.06 to 5.91) | 3.50 (0.03 to 354.84) | 0.18 (0.00 to 30.19) | 0.509 |
| MEK, Chemotherapy* | 2.86 (1.39 to 5.90) | 0.18 (NE) | 15.89 (NE) | 0.984 |
| CTLA-4/Chemo, Chemotherapy* | 3.05 (0.12 to 74.48) | 0.24 (NE) | 12.49 (NE) | 0.998 |
| BRAF, Chemotherapy | 2.62 (0.11 to 64.00) | 0.46 (0.01 to 25.96) | 5.65 (0.03 to 965.92) | 0.509 |
| PD-1, PD-1/CTLA-4* | 0.33 (0.01 to 8.15) | 2.57 (NE) | 0.13 (NE) | 0.998 |
| CTLA-4 low, PD-1/CTLA-4* | 0.34 (0.01 to 8.20) | 0.04 (NE) | 9.22 (NE) | 0.999 |
| CTLA-4 low, PD-1* | 1.01 (0.02 to 50.56) | 0.29 (NE) | 3.51 (NE) | 0.998 |
| CTLA-4 low, CTLA-4 high* | 0.34 (0.01 to 8.20) | 3.09 (NE) | 0.11 (NE) | 0.999 |
| BRAF, BRAF/MEK | 0.60 (0.25 to 1.45) | 1.10 (0.20 to 6.07) | 0.55 (0.08 to 3.75) | 0.541 |
| **Arthralgia** |  |  |  |  |
| PD-1, Placebo | 2.96 (0.31 to 28.35) | 5.55 (0.56 to 55.09) | 0.53 (0.02 to 13.34) | 0.702 |
| CTLA-4 high, Placebo | 5.03 (0.24 to 104.53) | 4.23 (0.28 to 63.09) | 1.19 (0.02 to 69.14) | 0.933 |
| BRAF/MEK, Placebo | 8.94 (0.48 to 165.52) | 8.34 (1.01 to 68.68) | 1.07 (0.03 to 39.27) | 0.97 |
| BRAF, Placebo | 35.00 (2.12 to 578.83) | 20.26 (2.56 to 160.56) | 1.73 (0.05 to 56.43) | 0.759 |
| PD-1, Chemotherapy | 0.96 (0.16 to 5.84) | 0.51 (0.02 to 10.56) | 1.86 (0.06 to 63.19) | 0.729 |
| MEK, Chemotherapy* | 1.42 (0.06 to 34.43) | 0.03 (NE) | 40.53 (NE) | 0.996 |
| CTLA-4/Chemo, Chemotherapy* | 3.05 (0.12 to 74.48) | 0.03 (NE) | 100.23 (NE) | 0.996 |
| BRAF, Chemotherapy | 4.64 (1.51 to 14.31) | 8.66 (0.31 to 243.94) | 0.54 (0.02 to 18.16) | 0.729 |
| PD-1, PD-1/CTLA-4 | 0.54 (0.07 to 4.01) | 1.30 (0.02 to 80.52) | 0.41 (0.00 to 41.12) | 0.706 |
| CTLA-4 low, PD-1/CTLA-4* | 1.02 (0.11 to 9.14) | 6.73 (0.01 to 5818.85) | 0.15 (0.00 to 287.23) | 0.624 |
| CTLA-4 high, PD-1 | 2.00 (0.18 to 21.93) | 0.59 (0.05 to 7.71) | 3.38 (0.10 to 113.46) | 0.497 |
| CTLA-4 low, PD-1 | 0.62 (0.05 to 7.19) | 16.44 (0.63 to 428.07) | 0.04 (0.00 to 2.57) | 0.128 |
| CTLA-4 low, CTLA-4 high | 5.03 (0.24 to 104.36) | 0.70 (0.04 to 13.71) | 7.14 (0.10 to 497.79) | 0.364 |
| BRAF, BRAF/MEK | 2.88 (1.50 to 5.54) | 2.69 (0.08 to 92.88) | 1.07 (0.03 to 39.26) | 0.97 |
| **Myalgia** |  |  |  |  |
| BRAF/MEK, Placebo | 2.98 (0.12 to 72.94) | 3.54 (0.11 to 109.79) | 0.84 (0.01 to 91.99) | 0.943 |
| BRAF, Placebo | 7.00 (0.36 to 134.82) | 5.90 (0.15 to 225.37) | 1.19 (0.01 to 129.67) | 0.943 |
| PD-1, Chemotherapy* | 0.32 (0.01 to 7.77) | 0.78 (NE) | 0.41 (NE) | 0.997 |
| BRAF, Chemotherapy* | 2.62 (0.11 to 64.00) | 1.45 (NE) | 1.81 (NE) | 0.998 |
| PD-1, PD-1/CTLA-4 | 3.77 (0.16 to 86.66) | 7.08 (0.05 to 1055.41) | 0.53 (0.00 to 209.32) | 0.836 |
| CTLA-4 low, PD-1/CTLA-4* | 2.97 (0.25 to 35.09) | 10.46 (NE) | 0.28 (0.00 to 4374.00) | 0.798 |
| CTLA-4 low, PD-1* | 0.54 (0.05 to 6.38) | 9.63 (NE) | 0.06 (0.00 to 841.46) | 0.556 |
| CTLA-4 low, CTLA-4 high* | 1.01 (0.06 to 16.02) | 0.32 (NE) | 3.12 (NE) | 0.999 |
| BRAF, BRAF/MEK | 1.98 (0.34 to 11.35) | 2.35 (0.03 to 183.16) | 0.84 (0.01 to 91.98) | 0.943 |
| **Diarrhea** |  |  |  |  |
| PD-1, Placebo | 1.31 (0.30 to 5.85) | 0.81 (0.35 to 1.85) | 1.63 (0.30 to 8.96) | 0.576 |
| CTLA-4 high, Placebo | 4.73 (2.42 to 9.25) | 8.90 (2.19 to 36.16) | 0.53 (0.11 to 2.51) | 0.425 |
| BRAF/MEK, Placebo | 3.97 (0.45 to 35.40) | 4.40 (0.92 to 21.01) | 0.90 (0.06 to 13.26) | 0.94 |
| BRAF, Placebo | 2.50 (0.49 to 12.76) | 1.40 (0.27 to 7.19) | 1.79 (0.18 to 18.05) | 0.622 |
| PD-1, Chemotherapy | 0.97 (0.34 to 2.80) | 0.92 (0.11 to 7.97) | 1.06 (0.10 to 11.66) | 0.965 |
| MEK, Chemotherapy* | 0.69 (0.14 to 3.45) | 1.56 (NE) | 0.44 (NE) | 0.998 |
| CTLA-4/Chemo, Chemotherapy | 16.26 (2.17 to 121.67) | 2.85 (0.05 to 162.11) | 5.70 (0.06 to 519.68) | 0.45 |
| BRAF, Chemotherapy | 1.40 (0.21 to 9.53) | 2.74 (0.43 to 17.32) | 0.51 (0.04 to 7.31) | 0.622 |
| PD-1, PD-1/CTLA-4 | 0.26 (0.13 to 0.52) | 0.35 (0.10 to 1.14) | 0.75 (0.18 to 3.13) | 0.691 |
| CTLA-4 low, PD-1/CTLA-4* | 0.82 (0.53 to 1.28) | 0.85 (0.12 to 6.04) | 0.97 (0.13 to 7.33) | 0.973 |
| CTLA-4 high, PD-1 | 6.13 (2.79 to 13.48) | 5.68 (2.82 to 11.40) | 1.08 (0.38 to 3.09) | 0.886 |
| CTLA-4 low, PD-1 | 2.90 (1.53 to 5.48) | 3.02 (1.38 to 6.60) | 0.96 (0.36 to 2.57) | 0.933 |
| CTLA-4 low, CTLA-4/Chemo | 0.90 (0.02 to 44.20) | 0.16 (0.02 to 1.55) | 5.70 (0.06 to 519.59) | 0.45 |
| CTLA-4 low, CTLA-4 high | 0.48 (0.30 to 0.78) | 0.59 (0.23 to 1.51) | 0.81 (0.28 to 2.31) | 0.692 |
| BRAF, BRAF/MEK | 0.44 (0.19 to 0.99) | 0.48 (0.04 to 6.25) | 0.90 (0.06 to 13.26) | 0.94 |
| **Nausea** |  |  |  |  |
| PD-1, Placebo | 0.99 (0.06 to 15.73) | 2.84 (0.38 to 21.13) | 0.35 (0.01 to 10.64) | 0.545 |
| CTLA-4 high, Placebo | 4.03 (0.45 to 35.88) | 1.25 (0.09 to 17.11) | 3.21 (0.11 to 96.96) | 0.502 |
| BRAF/MEK, Placebo | 8.94 (0.48 to 165.52) | 6.65 (0.75 to 58.64) | 1.34 (0.04 to 51.26) | 0.874 |
| BRAF, Placebo | 3.00 (0.12 to 73.29) | 4.73 (0.66 to 33.77) | 0.63 (0.01 to 27.03) | 0.812 |
| PD-1, Chemotherapy | 0.31 (0.08 to 1.18) | 0.19 (0.02 to 2.31) | 1.64 (0.10 to 27.60) | 0.73 |
| MEK, Chemotherapy* | 1.30 (0.26 to 6.48) | 0.02 (NE) | 70.24 (NE) | 0.992 |
| CTLA-4/Chemo, Chemotherapy | 0.58 (0.17 to 1.96) | 1.68 (0.04 to 63.36) | 0.35 (0.01 to 15.91) | 0.587 |
| BRAF, Chemotherapy | 0.60 (0.19 to 1.96) | 0.54 (0.02 to 12.91) | 1.12 (0.04 to 33.33) | 0.947 |
| PD-1, PD-1/CTLA-4 | 0.19 (0.04 to 0.82) | 0.92 (0.04 to 21.57) | 0.20 (0.01 to 7.20) | 0.38 |
| CTLA-4 low, PD-1/CTLA-4* | 0.38 (0.10 to 1.43) | 0.28 (0.00 to 33.14) | 1.35 (0.01 to 240.81) | 0.91 |
| CTLA-4 high, PD-1 | 0.33 (0.01 to 8.14) | 1.88 (0.33 to 10.81) | 0.18 (0.00 to 6.76) | 0.351 |
| CTLA-4 low, PD-1 | 1.38 (0.26 to 7.33) | 1.50 (0.19 to 11.77) | 0.92 (0.07 to 13.05) | 0.952 |
| CTLA-4 low, CTLA-4/Chemo | 0.30 (0.01 to 7.13) | 0.87 (0.10 to 7.44) | 0.35 (0.01 to 15.90) | 0.587 |
| CTLA-4 low, CTLA-4 high | 1.05 (0.22 to 4.98) | 1.38 (0.10 to 18.36) | 0.76 (0.04 to 15.55) | 0.859 |
| BRAF, BRAF/MEK | 0.57 (0.25 to 1.33) | 0.43 (0.01 to 14.74) | 1.34 (0.04 to 51.25) | 0.874 |
| **Rash** |  |  |  |  |
| PD-1, Placebo | 2.96 (0.09 to 92.35) | 1.03 (0.08 to 13.77) | 2.88 (0.04 to 214.09) | 0.631 |
| CTLA-4 high, Placebo | 13.08 (0.57 to 300.99) | 2.16 (0.12 to 38.77) | 6.07 (0.09 to 431.10) | 0.407 |
| BRAF/MEK, Placebo | 0.99 (0.05 to 20.96) | 3.43 (0.54 to 21.93) | 0.29 (0.01 to 10.28) | 0.496 |
| BRAF, Placebo | 4.67 (0.77 to 28.17) | 8.21 (0.63 to 107.72) | 0.57 (0.02 to 13.13) | 0.724 |
| PD-1, Chemotherapy | 1.56 (0.19 to 12.55) | 14.82 (0.65 to 340.04) | 0.11 (0.00 to 4.54) | 0.241 |
| MEK, Chemotherapy* | 13.10 (1.49 to 115.13) | 0.64 (NE) | 20.62 (NE) | 0.988 |
| CTLA-4/Chemo, Chemotherapy | 7.11 (0.28 to 178.42) | 6.84 (0.15 to 308.03) | 1.04 (0.01 to 152.30) | 0.988 |
| BRAF, Chemotherapy | 49.72 (2.37 to 1044.49) | 1.86 (0.06 to 55.56) | 26.73 (0.28 to 2559.42) | 0.158 |
| PD-1, PD-1/CTLA-4 | 0.13 (0.01 to 1.36) | 0.30 (0.01 to 8.14) | 0.44 (0.01 to 30.92) | 0.705 |
| CTLA-4 low, PD-1/CTLA-4* | 0.42 (0.10 to 1.82) | 0.04 (0.00 to 10.87) | 11.26 (0.03 to 4116.37) | 0.421 |
| CTLA-4 high, PD-1 | 2.79 (0.53 to 14.62) | 4.28 (0.50 to 36.44) | 0.65 (0.04 to 9.78) | 0.757 |
| CTLA-4 low, PD-1 | 1.89 (0.30 to 11.84) | 2.15 (0.27 to 16.92) | 0.88 (0.06 to 13.23) | 0.924 |
| CTLA-4 low, CTLA-4/Chemo | 0.90 (0.04 to 18.35) | 0.86 (0.02 to 45.79) | 1.04 (0.01 to 152.28) | 0.988 |
| CTLA-4 low, CTLA-4 high | 0.69 (0.11 to 4.21) | 0.50 (0.05 to 4.95) | 1.40 (0.07 to 26.26) | 0.823 |
| BRAF, BRAF/MEK | 2.12 (0.89 to 5.08) | 7.34 (0.23 to 236.15) | 0.29 (0.01 to 10.28) | 0.496 |

* All the evidence about these contrasts comes from the trials which directly compare them.

** P equal or less than 0.05 indicates a significant inconsistency between the direct effect and indirect effects.

AEs: adverse events; ALT: alanine aminotransferase; AST: aspartate aminotransferase; CI: confidence interval; Chemo: Chemotherapy; CTLA-4: cytotoxic T-lymphocyte-associated antigen-4 inhibitors; NE: not estimable; PD-1: programmed cell death protein 1 inhibitors;RR: relative risk.
